# MicroRNAs are deeply linked to the emergence of the complex octopus brain

**DOI:** 10.1126/sciadv.add9938

**Published:** 2022-11-25

**Authors:** Grygoriy Zolotarov, Bastian Fromm, Ivano Legnini, Salah Ayoub, Gianluca Polese, Valeria Maselli, Peter J. Chabot, Jakob Vinther, Ruth Styfhals, Eve Seuntjens, Anna Di Cosmo, Kevin J. Peterson, Nikolaus Rajewsky

**Affiliations:** ^1^ Laboratory of Systems Biology of Gene Regulatory Elements, Berlin Institute for Medical Systems Biology, Max Delbrück Center for Molecular Medicine in the Helmholtz Association, Hannoversche Str 28, 10115 Berlin, Germany.; ^2^ Centre for Genomic Regulation (CRG), Barcelona Institute of Science and Technology (BIST), Barcelona, Spain.; ^3^ Universitat Pompeu Fabra (UPF), Barcelona, Spain.; ^4^ UiT The Arctic University of Norway, Tromsø, Norway.; ^5^ SciLifeLab, Stockholm University, Stockholm, Sweden.; ^6^ Department of Biology, University of Naples Federico II, Naples, Italy.; ^7^ Dartmouth College, Hanover, NH, USA.; ^8^ School of Earth Sciences, University of Bristol, Bristol, UK.; ^9^ School of Biological Sciences, University of Bristol, Bristol, UK.; ^10^ Laboratory of Developmental Neurobiology, Department of Biology, KU Leuven, Leuven, Belgium.; ^11^ Department of Biology and Evolution of Marine Organisms, Stazione Zoologica Anton Dohrn, Naples, Italy.

## Abstract

Soft-bodied cephalopods such as octopuses are exceptionally intelligent invertebrates with a highly complex nervous system that evolved independently from vertebrates. Because of elevated RNA editing in their nervous tissues, we hypothesized that RNA regulation may play a major role in the cognitive success of this group. We thus profiled messenger RNAs and small RNAs in three cephalopod species including 18 tissues of the *Octopus vulgaris*. We show that the major RNA innovation of soft-bodied cephalopods is an expansion of the microRNA (miRNA) gene repertoire. These evolutionarily novel miRNAs were primarily expressed in adult neuronal tissues and during the development and had conserved and thus likely functional target sites. The only comparable miRNA expansions happened, notably, in vertebrates. Thus, we propose that miRNAs are intimately linked to the evolution of complex animal brains.

## Teaser

The ancestors of octopuses and squids have expanded their small RNA toolkit which enabled the evolution of complex brains.

## INTRODUCTION

Coleoid (soft-bodied) cephalopods (octopuses, squids, and cuttlefishes) have elaborate nervous systems both in terms of size and organization (*1*–*4*). Understanding the molecular mechanisms behind the evolution of the coleoid nervous system thus offers the opportunity to find general molecular design principles behind morphological and behavioral complexity in animals. Octopus (*5*) and squid (*6*) genomes do not show signs of whole-genome duplications, and the intronic architecture, as well as protein-coding content, was found to largely resemble those of other related invertebrates (*7*). Recently, it was shown that coleoids extensively use A-to-I RNA editing (*8*, *9*) mediated by ADAR enzymes (“adenosine deaminases acting on RNAs”) (*10*) to recode their neuronal transcriptomes. Because extensive editing is not abundant in other mollusks including *Nautilus*, a cephalopod and the living sister group of the coleoids with a simpler nervous system, this process has been hypothesized to drive the cognitive success of coleoids (*9*), perhaps by providing a mechanism to expand and regulate the coding repertoire of mRNAs. However, it is difficult to explain the evolution of complex heritable traits by the actions of a single trans-acting factor, and it has been proposed that the editing phenomena in coleoids are mainly nonadaptive [(*11*), but see (*12*)]. Because ADARs interact and regulate many classes of RNAs [for example, the silencing of transposon RNA (*13*), the biogenesis of circular RNAs (circRNAs) (*14*), and defense against viral RNAs (*15*)], we hypothesized that posttranscriptional regulation of RNA in general is potentially linked to the evolution of the complex nervous system of the coleoid cephalopods.

## RESULTS

**Fig. 1.**
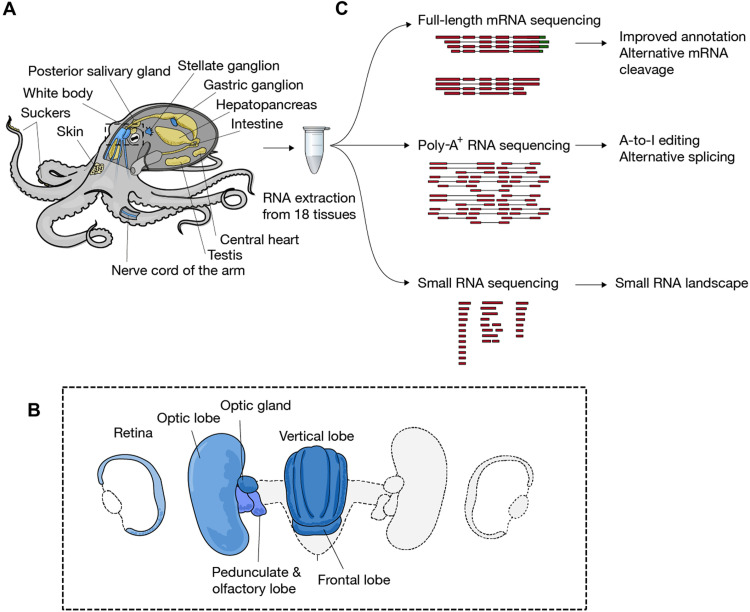
RNA profiling of the common octopus *O. vulgaris*. (**A**) Schematic representation of tissues sampled in the study. Neuronal and non-neuronal tissues are colored in blue and yellow, respectively. Inset (**B**): Brain and surrounding structures. (**C**) Main sequencing methods and computational analyses used in this study.

Thus, we first systematically quantified major modes of posttranscriptional regulation across 18 tissues of adult octopus (Fig. 1, A and B, and tables S1 and S2). For each mode of regulation, we also checked whether A-to-I editing adds complexity to regulation. Briefly (see Supplementary Text for an in-depth presentation), we combined mRNA shotgun and two full-length mRNA sequencing methods [Iso-Seq from PacBio and full-length poly(A) and mRNA sequencing (FLAM-seq) (*16*)] to produce a high-quality dataset of 56,579 mRNA isoforms covering 10,957 reference genes (data file S1). In both neuronal and non-neuronal tissues, most of the A-to-I editing occurred in the introns and 3′ untranslated regions (3′UTRs) of mRNAs, consistent with the elevated presence of ADAR substrates (hairpin structures) in these regions compared to coding sequences (fig. S1). We found that alternative splicing was highest in neural tissues, as expected, and that A-to-I editing very rarely altered splice sites (fig. S2 and table S3). CircRNAs were expressed at overall low levels, consistent with the reported repression of circRNA biogenesis by ADAR (*14*, *17*). When analyzing polyadenylate [poly(A)] tails with FLAM-seq, we found that poly-A tails from the octopus testes were significantly shorter than in any other tissue and, unexpectedly, contained a high fraction of guanosines, a phenomenon not seen in other species (Supplementary Text and fig. S3). 3′UTRs had a median length of around 350-380 nucleotides (nt), longer than in well-studied invertebrate model systems.

In summary, the transcriptome of *Octopus vulgaris* does not show major departures from other invertebrates in terms of alternative splicing diversity and rates, as well as in mRNA cleavage and polyadenylation. The most outstanding feature was 3′UTR length, and we thus turned our attention to microRNAs (miRNAs) that are known to bind 3′UTRs with these interactions showing dynamic patterns over evolutionary history (*18*).

### An expansion of the miRNA repertoire in coleoid cephalopods

When annotating miRNAs from small RNA sequencing data, a fundamental problem is the detection of a large number of lowly expressed small RNAs that are likely background products of the miRNA biogenesis pathway without functional importance (*19*). To focus only on robustly supported miRNA genes, we independently annotated the miRNAs from all 18 *O. vulgaris* tissue datasets, as well as from a whole-body small RNA dataset from a second octopus species, *Octopus bimaculoides*, which split from *O. vulgaris* ~50 million years ago (*20*) (Materials and Methods and Supplementary Text) following standard miRNA annotation criteria (*21*, *22*). To minimize the proliferation of false-positive miRNAs, a novel miRNA in one octopus species had to be present in the second before it was considered a novel miRNA family. With this criterion in place, we identified a total of 164 miRNA genes grouped into 138 miRNA families in *O. vulgaris*, and 162 miRNA genes grouped into the same 138 families in *O. bimaculoides.* We stress that this is likely an underestimate of the number of octopus miRNA genes as our sequencing data are incomplete and we are missing miRNAs that may have evolved during the past 50 million years in one or the other octopus species. However, we recovered 46 of 48 miRNA families expected to be present in octopus given its phylogenetic position (Materials and Methods). Two families (MIR-1989 and MIR-242) were not found in the expression data or genomes from both octopus species (*6*) and appear to be true losses in this lineage. In total, 43% (70 of 164) of predicted miRNA genes in *O. vulgaris* could be assigned to known miRNA families described in other animals. The remaining 94 genes were assigned to 90 novel miRNA families, none of which co-occur with another novel family on any genomic scaffold (Mirgenedb.org).

**Fig. 2.**
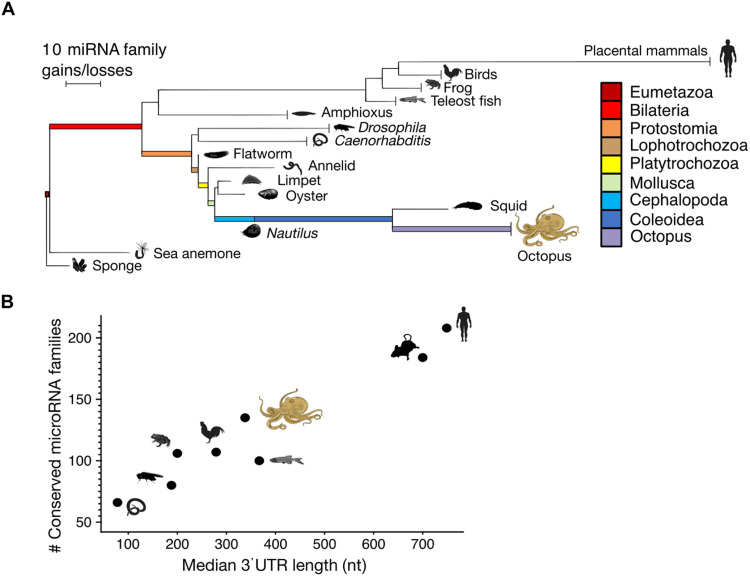
Expansion of the miRNA repertoire in cephalopods. (**A**) Phylogeny of several animal groups with the branch lengths between nodes, or from a node to an extant species, reflecting the gains of miRNA families minus the losses (Materials and Methods). Vertical lines at the end of the branches indicate the shared complement of the indicated taxon as deposited in MirGeneDB (*21*); the other branches lead to single species (sponge: *A.* *queenslandica*; sea anemone: *N. vectensis*; flatworm: *S. mediterranea*; annelid: *C. teleta*; oyster: *C. gigas*; limpet: *L. gigantea*). (**B**) Number of miRNA families (excluding species-specific novel families) versus median 3′UTR length in selected animals. For instance, “Human” represents the number of miRNA families annotated in genus *Homo*. Median lengths of 3′UTRs were computed from genome annotations (Materials and Methods).

To more precisely determine the evolutionary origin of these 90 novel miRNA families, we sequenced a whole-body small RNA library from the bobtail squid *Euprymna scolopes*, as well as searched the recently published *Nautilus* genome (*23*) for miRNA precursor sequences and annotated their respective miRNA complements. Both MIR-1989 and MIR-242 were again not found in the expression data or in the genome of the bobtail squid but was found in the genome of *Nautilus*, and thus, these miRNAs were lost in the coleoid lineage. Of the 90 novel miRNA families identified in octopus, 12 were found in the genome of *Nautilus* and the squid and thus represent the cephalopod miRNA set (Fig. 2A). An additional 43 novel miRNA families are shared between the two octopus species and squid and thus represent miRNA families that emerged in coleoid lineage. Last, an additional 35 miRNA families are restricted to the *Octopus* lineage.

This marked expansion of the miRNA gene repertoire leading to the octopus lineage is the largest gain of shared miRNA families known within the invertebrates, and the total number of miRNA families in the octopus genome (138) is on par with that found in vertebrates (minus placental mammals) including chicken (107 families), African clawed frog (106), or zebrafish (100) (Fig. 2A). An evolutionary expansion of the number of miRNAs is generally linked to an expansion in the length of 3′UTRs (*24*), the targets of miRNAs. When using our measured 3′UTR lengths in octopus and graphing the number of conserved miRNAs versus median 3′UTR length for octopus and other species, the octopus data fit nicely into the expected position (Fig. 2B).

### Novel miRNAs are specifically expressed in neural tissues and during development

**Fig. 3.**
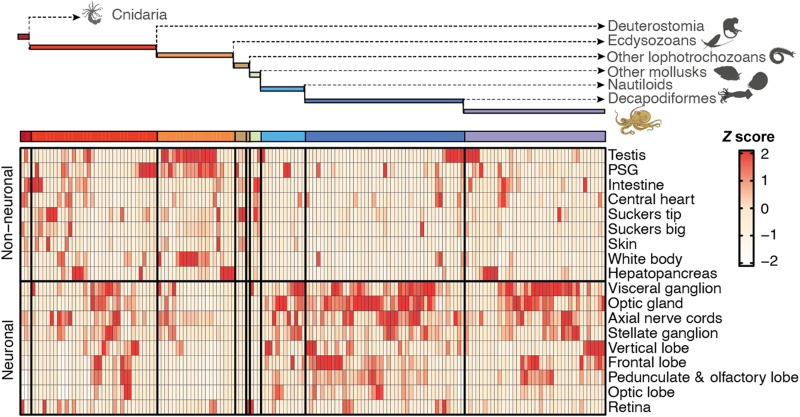
Novel, conserved octopus miRNAs are specifically expressed in neuronal tissues. A simplified phylogenetic tree showing the number of miRNAs that evolved from the time bilaterians split from cnidarians to the last common ancestor of the two considered *Octopus* species. Color code as in Fig. 2. For each miRNA (columns), its expression distribution across tissues (rows) in both neural and non-neural tissues and the corresponding *Z* scores were computed. Columns within each bin were hierarchically clustered on the basis of the *Z* scores (extended version: fig. S3A). PSG, posterior salivary gland.

We next investigated tissue expression patterns of octopus miRNAs as a function of their evolutionary age. Deeply conserved bilaterian miRNAs recapitulated known tissue expression patterns (table S5) (*25*). Most of the cephalopod and coleoid-specific miRNAs were expressed, as expected, at overall lower levels than older miRNAs (fig. S4) (*26*, *27*). However, novel miRNAs were primarily expressed in the nervous tissues of the animal (Fig. 3). Of the 43 miRNAs of coleoid origin, 34 have their maximum of expression in one or more neural tissues. In these tissues, they are expressed, on average, at 13 times higher levels than in non-neuronal tissues (figs. S4, B and C, and S5). The sampled non-neuronal tissue with the highest coverage (“suckers tip,” 50 million reads; 53.6%) had a lower proportion of captured novel miRNAs than the neuronal tissue with the lowest coverage (“pedunculate and olfactory lobe”, 30 million reads; 57%). Thus, the fact that the novel miRNAs are most specifically expressed in neural tissues is not due to a potential tissue sampling bias (Supplementary Text and fig. S5).

**Fig. 4.**
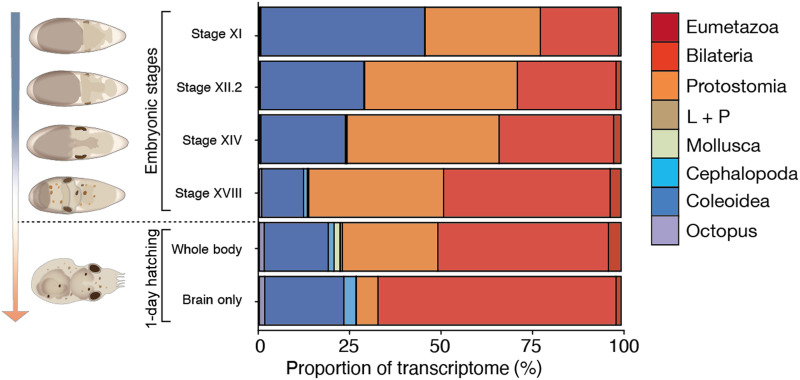
Novel, conserved octopus miRNAs are highly expressed during development. Proportions of miRNA transcriptomes dedicated to miRNAs of different phylogenetic nodes of origin. Samples were obtained by developmental stage of *O. vulgaris* (**65**). These samples cover the organogenic stages of *O. vulgaris* development (stage XI to stage XVIII) when most of the embryonic growth occurs, as well as the whole body and brain of 1-day-old paralarvae when the growth of the larval brain commences. “L + P” refers to the collective miRNAs that evolved in lophotrochzoans and platytrochozoans (see Fig. 2).

If these novel miRNAs contributed to the evolution of coleoid brains, then they would be expected to be expressed during neural development. To test this prediction, we profiled small RNA expression at the late stages of *O. vulgaris* development before hatching. Moreover, immediately after hatching, we sequenced small RNAs from whole-body hatchlings and isolated brains (Fig. 4). Novel coleoid miRNAs were robustly expressed during development, and octopus embryos at developmental stage XI had the highest relative proportion (~45%) of an miRNA transcriptome devoted to coleoid miRNAs of all 22 tissues sequenced in this study (fig. S6). Together, our data suggest that novel coleoid miRNAs contribute brain development in octopus.

### Target sites of novel miRNAs are conserved

**Fig. 5.**
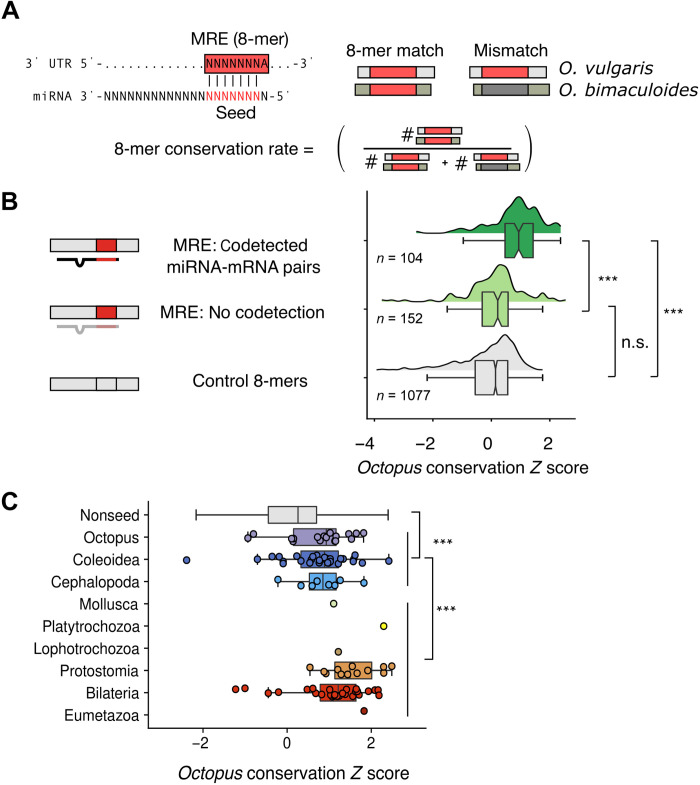
Target sites of novel miRNAs are conserved and coexpressed with the respective miRNA. (**A**) Definition of MREs (or “8-mer”) and their evolutionary conservation. The 8-mer conservation rate is defined as the percentage of occurrences in 3′UTRs, where a particular 8-mer (red) is matched by exactly the same 8-mer at the same position in the aligned orthologous 3′UTR. (**B**) Shown here, for novel octopus miRNAs (conserved between *O.* *vulgaris* and *O.* *bimaculoides*), is the MRE conservation rate in units of a standard *Z* score. Coexpression is defined as an mRNA with an MRE and the respective miRNA codetected in at least one tissue at 10 and 100 counts per million, respectively (Materials and Methods). Coexpressed miRNA-MRE pairs are statistically more highly (*P* < 0.001) conserved than non-coexpressed pairs or control 8-mers which were not related to any MRE in the octopus. (**C**) As expected, MRE conservation rates are higher for evolutionarily older miRNA families. In (B) and (C), statistical significances: Mann-Whitney *U* test with Bonferroni correction for multiple hypothesis testing (n.s., *P* > 0.05 and ***P < 0.001).

If miRNA target sites are conserved across sufficiently large evolutionary distances, then it is likely that these sites are functionally important. Thus, to show that the shared miRNA complement of the two *Octopus* species is functional, we asked whether their target sites are conserved between these two species. To this end, we defined “miRNA response elements” (MREs) as an octamer starting with adenosine, followed by a heptamer Watson-Crick complementary to positions 2 to 8 of the miRNA as these MREs generally mediate the strongest regulatory effect when bound by the respective miRNA (Fig. 5A) (*28*, *29*). Predicted MREs shared between the two octopus species showed higher conservation rates compared to the control 8-mers (Fig. 5B, Materials and Methods, and data file S2). As expected (*30*), this signal disappeared when miRNA: target pairs were not coexpressed (Fig. 5B, Materials and Methods, and data file S2), strongly suggesting that the conservation of MREs is caused by the functional interaction between the miRNA and the MRE in the respective tissues. Last, MREs of phylogenetically younger miRNA families were, on average, less conserved than MREs from older miRNA families (i.e., miRNAs of protostome or bilaterian origin) (Fig. 5C), consistent with their generally lower expression levels and potentially lower selection pressure to maintain their target sites (*31*). Overall, we conclude that the novel octopus miRNAs are functional and exert function, at least in part, by canonical seed-pairing mechanism.

### In octopus, A-to-I editing is decoupled from miRNA function

We asked whether A-to-I editing is potentially modulating miRNA function in the octopus. This could occur by (i) editing the miRNAs themselves and/or (ii) editing miRNA target sites in 3′UTRs (destroying or creating them). Briefly, we found no evidence for any functionally important editing of miRNAs (Supplementary Text). We could detect only five miRNAs with an estimated A-to-I editing frequency in the seed sequence above 1% (but never more than 4.8%) (fig. S7 and Materials and Methods). Similarly, we found that A-to-I editing events with the potential to destroy miRNA target sites (MREs) happen rarely. Of 10,053 MREs conserved between the two octopus species and having sufficient RNA sequencing (RNA-seq) coverage, only 39 (0.3%) harbored editing events (Materials and Methods and Supplementary Text). Last, we found no higher conservation for 8-mers potentially becoming an MRE by A-to-I editing compared to control sequences (fig. S8 and Supplementary Text). This suggests that de novo creation of MREs or disruption of existing MREs by editing is, if existent, a rare phenomenon.

## DISCUSSION

Coleoid cephalopods are unusual among invertebrates in having a nervous system comparable to the central nervous system of vertebrates, at least in terms of the neuronal number and anatomical specialization (*3*) and hence in terms of its complexity (*32*, *33*). Nonetheless, the complexity of the coleoid nervous system belies the generality of its protein-encoding genomic content, in particular its set of transcription factors (table S2). Aside from independent expansions of the C2H2 zinc finger–encoding and protocadherin-encoding genes in the squid and octopus lineages, the octopus has a canonical repertoire of transcription factors similar to other lophotrochozoans (*5*, *6*). Coupling this generalized protein-encoding repertoire with the reported elevated rates of A-to-I editing in coleoid neural tissues (*8*, *9*) led us to hypothesize that RNA regulation in general might be involved in driving an apparent increase in the complexity of the coleoid nervous system. Our data and analyses argue that in terms of alternative splicing diversity and rates (including back-splicing that generates circRNAs), as well as mRNA cleavage and polyadenylation patterns, there is no major departure from other invertebrates. Further, we find no evidence for substantial editing in miRNA seed sequences nor in potential target sites either in the abrogation of a genetically encoded site or in the creation of a newly relevant site (figs. S7 and S8). Furthermore, a recent study in *O. bimaculoides* and squid *Doryteuthis pealeii* reports no enrichment of A-to-I editing in any particular protein domain genome-wide with the vast majority of editing events found outside of coding regions (*34*). Of course, A-to-I editing may still be functionally important in individual cases (*35*), but the main function of this process in coleoids remains elusive.

On the other hand, a clear distinction in RNA regulation between coleoid cephalopods and all other known invertebrates is reflected in the marked expansion of their miRNA repertoire. The conservation of more than 50 miRNA loci in both the squid and octopus lineages since they diverged from one another nearly 300 million years ago (*20*) coupled with the 3′UTR (Fig. 2B), miRNA expression (Figs. 3 and 4), and target site (Fig. 5) analyses discussed above all strongly suggest that these miRNAs are functionally important during the development of the coleoid nervous system. In stark contrast to *Octopus* that evolved 90 novel miRNA families since its last common ancestor with the oyster *Crassosstrea*, the genus *Crassostrea* evolved only five novel miRNA families over the same span of geological time (*36*) as assessed through comparable levels and samples of small RNA sequencing data. Like in virtually all other increases to a miRNA repertoire, both the source and evolutionary pressures for the rise of these novel miRNA loci are not known; whole-genome duplications can be ruled out (*5*, *6*), and scenarios may apply where novel miRNAs arise from the extensive genomic reorganizations found in coleoid taxa (*5*, *37*). Whatever their source, once under selection, miRNAs in general are believed to improve the robustness of the developmental processes (*38*–*42*), increasing the heritability of the interaction (*43*–*45*), which might then allow for the evolution of new cell types (*46*) and ultimately morphological and behavior complexity (*32*, *47*). With respect to the development of the nervous system, we note that at least in vertebrates, miRNAs are known to have highly complex expression patterns with, for example, miRNA transcripts localized to the synapse and modulating their function (**48**). Although it remains to be seen whether these types of pathways operate in coleoids, the notable explosion of the miRNA gene repertoire in coleoid cephalopods may indicate that miRNAs and, perhaps, their specialized neuronal functions are deeply linked and possibly required for the emergence of complex brains in animals.

## MATERIALS AND METHODS

### Tissue dissection and RNA extraction

Adult specimens of *O. vulgaris* (body weight of 800 ± 50 g, mean ± SD) were collected from the Bay of Naples (Italy) and transferred to the Department of Biology, University of Naples Federico II (Italy). The research was approved following the European Directive 2010/63 EU L276, the Italian DL. 4/03/2014, no. 26, and the ethical principles of Reduction, Refinement, and Replacement (project no. 608/2016-PR-17/06/2016; protocol no. DGSAF 0022292-P-03/10/2017). Samples (*n* = 8) were anesthetized by isoflurane insufflation (*49*), and tissues were dissected under sterile conditions following institutional guidelines. Tissues selected were as follows: axial nerve cords, central heart, vertical and frontal lobes, hepatopancreas, ink sac, intestine, optic gland, optic lobe, testes, pedunculate lobe and olfactory lobe, posterior salivary glands, retina, skin, stellate ganglion, suckers at the base and the tip of the arm, visceral (gastric) ganglion, and white body.

Collected samples were snap-frozen in liquid nitrogen and immediately put in TRIzol and then stored at −80°C. The RNA has been extracted from the tissues using Direct-zol^TM^ RNA/Miniprep (Zymo Research), following the manufacturer’s protocol. The quality of RNA has been accessed with Bioanalyzer and only samples with intact RNA have been kept for library preparation.

A juvenile individual of *O. bimaculoides* (mantle size, ~ 2.5 cm) was obtained from the National Resource Centre for Cephalopods (Galveston, Texas) on 24 September 2009. The specimen was shipped alive to Dartmouth College. The specimen was euthanized immediately by submersion directly into liquid nitrogen in a large mortar held in an ice bucket filled with dry ice. When completely frozen after a few minutes, it was homogenized into a powder using a pestle. About 5 g of homogenized powder was transferred to a 50-ml Oak Ridge screw cap centrifuge tube and mixed with TRIzol for total RNA extraction using the standard protocol with glycogen added during the precipitation step (Invitrogen, Carlsbad).

Strings of eggs of *O. vulgaris* were obtained from the Instituto Español de Oceanografía (Tenerife, Spain). Embryos were incubated in a standalone system at KU Leuven, Belgium and collected at different developmental time points (St XI, XII.2, XIV, XVIII, and 1-day-old paralarvae). Embryos were dechorionated and the yolk was removed. Paralarval brains were dissected as described before (**46**). RNA was extracted from a pool of embryos or brains using Tri-reagent (Invitrogen) and the Qiagen Micro kit (Qiagen). All experiments involving hatchlings were approved by the ethical committee (permit P080/2021).

### Poly-A^+^ mRNA library preparation and sequencing

Poly(A)^+^ RNA-seq libraries were prepared using the TruSeq Stranded mRNA Kit (Illumina) according to the manufacturer’s instructions. Libraries were sequenced on a NextSeq 500 device at 1 × 76 cycles.

### Total RNA library preparation and sequencing

Three hundred nanograms of total RNA per sample was first depleted of ribosomal RNA (rRNA) using the RiboCop rRNA Depletion Kit (Lexogen, #144) according to the manufacturer’s instructions. The rRNA-depleted samples were then processed with the TruSeq mRNA stranded kit from Illumina. Libraries were then sequenced on a NextSeq 500 device at 2 × 76 cycles (paired end).

### Full-length mRNA library preparation and sequencing

Two full-length mRNA sequencing approaches used in this study have their own strengths and weaknesses and thus are complementary. In particular, FLAM-seq is biased toward shorter molecules, generating libraries of 1.5-kbp median length. Iso-Seq, on the other hand, is susceptible to internal priming. These artifacts arise when oligo-dT primer aligns to an A-rich sequence inside mRNA instead of a poly(A) tail. The sequencing reads arising in result appear truncated from the 3′ end and may be misinterpreted as alternative isoforms. FLAM-seq is insensitive to such an artifact as it replaces oligo-dT priming with oligo-dC priming onto an enzymatically added 3′ guanosine/inosine anchor, and thus, the sequenced mRNAs include the entire, nontemplated poly(A) tail, which flags a detected mRNA 3′ end as bona fide. To produce a comprehensive annotation of the *O. vulgaris* transcriptome, we therefore combined FLAM-seq with Iso-Seq, with the addition of a size-selection step for enriching long transcripts that are underrepresented in FLAM-seq data. We reasoned that this strategy would combine the capacity of Iso-Seq to generate extremely long complementary DNA molecules with the high accuracy of FLAM-seq in retrieving bona fide 3′ ends of mRNA. For FLAM-seq library generation, the following detailed protocol was first applied to 4 μg of RNA from hepatopancreas: https://protocolexchange.researchsquare.com/article/pex-398/v1. On this sample, 16, 18, 20, or 22 polymerase chain reaction (PCR) cycles were performed. The library profiles were checked on a bioanalyzer high-sensitivity DNA assay and concluded that 20 cycles yielded the best result in terms of library size and quantity. The complete protocol with 20 PCR cycles was therefore applied to 4 μg of the six samples listed in table S1, and the generated libraries were barcoded at the SMRTbell adapter ligation step and multiplexed on six Sequel SMRTcells in total.

For Iso-Seq, the Iso-Seq Express 2.0 workflow (PacBio) was applied to 500 ng of RNA from each of the six samples listed in table S1, using barcoded PCR primers for 14 cycles of PCR amplification, and performing size selection after amplification with ProNex beads (Promega #NG2001), to enrich transcripts larger than 3 kb. Iso-Seq libraries were then also multiplexed and sequenced on six Sequel SMRTcells in total. As indicated in table S1, an additional FLAM-seq library and Iso-Seq library were generated in a second instance and again sequenced on one SMRTcell.

### Processing of PacBio SMRT data

To obtain full-length nonchimeric reads (FLNCs) from Iso-Seq sequencing data, isoseq3 pipeline (https://github.com/PacificBiosciences/IsoSeq) has been used without a polishing step. We decided to skip a polishing step to increase the sensitivity for low-abundance transcripts. To obtain FLNC equivalents from FLAM-seq data, FLAMAnalysis pipeline has been used (*16*). In brief, the sequencing reads have been mapped to the genome using STARlong aligner (*50*) using default parameters. Next, poly(A) tails have been identified and trimmed from each read. We have used the resulting reads as an input for the next step.

### Isoform reconstruction from FLNC reads

For Iso-Seq, FLNC reads have been mapped to the *Octopus sinensis* genome using minimap2 (*51*):

minimap2 -ax splice:hq -t {threads} -uf --secondary = no -C5 {params.genome} {input} > {output}.

Next, the putative transcripts have been assembled for each tissue using TAMA Collapse (*52*):

python tama_collapse.py -s {input} -f {genome} -x no_cap -e common_ends -c 99 -i 85 -a 10 -m 10 -z 20 -sjt 10 -lde 5 -log {params.log}.

FLAM-seq reads from FLAMAnalysis pipeline have been mapped to the genome with minimap2 using the same settings as above. As FLAM-seq provides precise resolution of the cleavage sites, TAMA Collapse has been used with a lower “three_prime_threshold” of 20 bp and a five prime threshold has been increased to 1000 bp, effectively leading to a collapse of all reads mapping to the same cleavage site in the genome.

Isoforms from individual libraries have been merged with TAMA merge:

tama_merge.py -m 10 -e common_ends -f {merge_file} -z 50 -a 10 -p {out_prefix} -d merge_dup

The merging resulted in 301,270 transcript models (86,394 if using previously polished models). In the merge file, higher weight has been given for FLAM-seq isoforms in determining 3′ end positions of the isoforms, and, conversely, higher weight has been given to Iso-Seq–derived isoforms in determining 5′ ends of the transcripts.

SQANTI2 tool (*53*) has been used to classify obtained isoforms with respect to the existing *O. sinensis* genome annotation:

python squanti2.py --skipORF --gtf --geneid --polyA_motif_list {polya_list} -t {threads} -n {params.chunks} -d {params.sqanti2

_out} -e {input.exp.} -c {splice} {isoforms_gtf} {genome_annotation} {genome_fasta}

Most of the isoforms supported by Iso-Seq data displayed elevated proportion of adenosines in the downstream genomic sequence, thus suggesting their probable origin due to internal priming.

Putative models have been filtered using the following criteria:

1) All transcripts with putative reverse template switch artifacts (as defined by SQANTI2) have been filtered out.

2) Full-splice match (FSM) transcripts have been retained only if the 3′ end was reliable (<6 A’s in 10-bp downstream genomic sequence).

3) Non-FSM transcripts have been retained only if:

a) 3′ end was reliable: Either polyadenylation signal (PAS) present or <6 A’s downstream.

b) Noncanonical junctions were supported by at least five uniquely mapping reads.

c) At least two reads were compatible with the isoform across all tissues.

4) Fusion transcripts have been removed from the annotation entirely.

5) Intergenic transcripts have been retained only if:

a) There was an open reading frame ≥ 100 GAA predicted.

b) The model was supported by at least five reads.

c) The model was supported by conventional RNA-seq reads.

d) The model was multi-exonic.

e) 3′ end was reliable (>6 A’s in 10-bp downstream sequence).

f) Passing splice junction support (vis above).

6) All antisense transcripts have been filtered.

7) Last, novel genes have been considered only if at least one of the associated transcripts:

a) Is multi-exonic.

b) Has splice junctions that are supported (vis above).

c) Is supported by either FLAM-seq method only or both.

d) Is supported by conventional short-read RNA-seq.

This filtering resulted in 59,579 mRNA isoforms associated with 10,957 reference genes.

These isoforms have been added to original *O. sinensis* genome annotation (GCF_006345805.1_ASM634580v1). All the original isoforms with FSM isoforms from the newly predicted set have been removed. The genome annotation is available as data file S1.

### Annotation of alternative splicing events

A Bioconductor SplicingGraphs (v 1.26.1; https://bioconductor.org/packages/release/bioc/html/SplicingGraphs.html) package has been used to count types of the alternative splicing events in the genome annotation obtained above by constructing and parsing splice graphs at each genomic locus as described in package vignette (https://bioconductor.org/packages/release/bioc/vignettes/SplicingGraphs/inst/doc/SplicingGraphs.pdf).

### Editing-generated splice sites

RNA-seq data from representative protostome animals (* Octopus bimaculoides* PRJNA285380, *Nautilus pompilius* PRJNA614552, *Crassostrea gigas* PRJNA146329, and *Capitella teleta* PRJNA379706) to corresponding genomes using STAR aligner (*50*) with the following parameters:

--alignIntronMin 20 --alignIntronMax 1000000 --alignSJoverhangMin 8

To make the datasets comparable, all sequencing reads have been trimmed to 50 bp and mapped to the respective genomes. The catalogs of splice sites provided by STAR aligner have been used to count the types of splice sites according to the intronic sequence.

To compare alternative splicing rates across different tissues, we have used approach from previous comparative studies of alternative splicing (*54*, *55*).

### Exon-skipping rates

Nonredundant exon triplets were extracted from the genome annotation .gff files using custom Python scripts. For each such triplet, three junctions were generated by concatenating to 42 bp from the upstream and downstream exons:

1) Exon1-exon2 (E1E2).

2) Exon2-exon3 (E2E3).

3) Exon2-exon3 (E1E3 an exon-skipping event).

An effective mappability of each junction has been calculated by extracting 50-mers (max, 35) and mapping them back using bowtie to the set of all junctions. Only the triplets with all three junctions having mappability ≥20 have been kept for the downstream analysis. In the following analysis, the number of reads mapping to the junction has been adjusted by multiplying the read counts by 35/[effective mappability]. For the exon-skipping rate (rES) analysis, only the reads not mapping to the genome have been used (i.e., those reads unmappable to the reference genome with bowtie). rES was then defined for each triplet as follows

$$\frac{N_{E1E3}}{(N_{E1E2}+N_{E2E3})/2+N_{E1E3}}$$

where *N_i_* represents mappability adjusted number of reads mapping to a junction *i*.

### Intron retention rates

Intron retention rates (rIRs) have been computed in a similar way but for the triplets consisting of the first bracketing exon (E1), intron (I), and the second bracketing exon (E2). rIR was then defined for each triplet as follows

$$\frac{N_{E1I}+N_{IE2}/2}{(N_{E1I}+N_{IE2})/2+N_{E1E2}}$$

### ES (IR) rate comparison across tissues

For every tissue, the triplets having at least five mapping reads to the major junctions (E1E2 + E2E2 for ES; E1I + IE2 for IR) and a minimal mappability of all three junctions have been retained. Then, 1000 triplets from this set have been chosen randomly and exactly 25,000 reads have been mapped to the junctions. Then, the rES (rIR) rate were determined as described above. This approach has been repeated 100 times for every tissue in the dataset.

### Annotation of circRNAs

FindCirc2 (https://github.com/rajewsky-lab/find_circ2) (*56*) has been used to annotate circRNAs from the total RNA-seq data. Putative circRNA predictions have been rigorously filtered to ensure high evidence of the backsplice junctions without any other explanations for the read mapping (such as trans-splicing events):

WARN_OTHER_CHROM_MATE==0 and WARN_OUTSIDE_SPLICE_JUNCTION==0 and SUPPORT_INSIDE_MATE>=5 and WARN_OUTSIDE_MATE<5 and WARN_OUTSIDE_SPLICE_JUNCTION==0 and WARN_UNRESOLVED_EXTRA_ BACKSPLICE==0 and WARN_UNRESOLVED_LINSPLICE==0.

Last, only back-splice junctions corresponding to known splice donor and acceptor sites have been kept.

### 3′UTR annotation with FLAM-seq

To annotate 3′UTRs in the octopus genome, we have used FLAM-seq data as this method avoids artifacts caused by internal priming. Each FLAM-seq read comes from an individual mRNA molecule (FLAM-seq uses unique molecule identifiers) and contains its full sequence and the sequence of the poly(A) tail. First, the reads were processed by a FLAMAnalysis pipeline (https://github.com/rajewsky-lab/FLAMAnalysis) to estimate the start position of a poly(A) tail and trim it from the sequence. Next, the resulting reads have been mapped to the genome using minimap2 (v 2.17-r941) using the parameters “--cs -ax splice:hq -uf --secondary=no -C5”, and the 3′-most position of each read has been recorded, generating ~110,000 initial putative cleavage sites. The tags closer that 20 base pairs have been merged, and their counts have been assigned to the most implicated site in the cluster. Then, the clusters were merged if separated by less than 40 nt (fig. S3A). Some genes may be missing from a genome annotation. To prevent sampling from these genes, which would induce misassignment of the FLAM-seq tags, we kept putative cleavage sites only if:

1) There is a continuous short RNA-seq coverage of at least five reads from the FLAM-seq tag to a stop codon of an upstream gene, or

2) At least one read assigned to the cluster overlaps the stop codon of the gene the cluster is assigned to.

A total of 40,949 putative cleavage sites have passed the filtering above and were included in the final dataset. This final set of cleavage sites contains 16,573 FLAM-seq clusters assigned to 7593 genes (fig. S3A).

### Sequence profiles around cleavage sites

The sequence compositions around cleavage sites for *Drosophila melanogaster* and *C. elegans* (fig. S3C) were obtained by extracting the regions around 3′ ends of gene models (both *D. melanogaster* and *C. elegans* genome annotations were obtained from Ensembl Metazoa; *C. elegans* genome assembly WBcel235; *D. melanogaster* genome assembly BDGP6.32).

### Polyadenylation signals

To determine PASs in the 3′UTRs, we queried the sequence upstream of the cleavage sites (50 nt) for a presence of PASs described in other metazoans. In cases of multiple PASs occurring in the sequence, the one that is more abundant in the whole dataset has been selected.

### 3′UTR and poly(A) length and composition in the tissues

For each gene, a mean distance from the FLAM-seq tags to the annotated stop codon has been computed with pooling FLAMseq reads from all tissues. The genes have been grouped on the basis of tissue expression pattern (whether the gene shows enriched expression in the nervous tissues) and on the basis of whether or not multiple 3′UTRs have been detected.

### A-to-I editing detection

To call editing events, short-read RNA-seq data generated in this study were mapped to the genome with STAR aligner (*50*). Alignment files were filtered to retain only uniquely mapping reads. The mismatches between the genome assembly and the alignments were called with bcftools (*57*). The same approach was used for DNA sequencing (DNA-seq) data from an *O. vulgaris* genome assembly project (*58*). Variants occurring in RNA-seq data but not in DNA-seq have been retrieved with bcftools isec. All genomic loci with more than 10 mapping reads of which at least 3 contain guanosine instead of adenosine were considered editing sites. This approach led to identification of 68,338 putative editing sites.

### A-to-I editing index

Editing index has been computed as described in (*59*). Briefly, for every genomic feature, the total numbers of adenosines and guanosines sequenced at all positions with reference A’s were determined. Then, an editing index was computed as the number of guanosines divided by a total number of sequenced adenosines.

### Small RNA library preparation and sequencing

#### O. vulgaris

Small RNA sequencing libraries were prepared using two different kits: The first one is SMARTer smRNA-Seq kit for Illumina from Clontech according to the manufacturer’s instruction using 10 ng of total RNA; the libraries were pooled together with 20% phix and sequenced on the NextSeq 500, 1 × 51. The second kit is the TruSeq Small RNA Kit from (Illumina). The libraries were prepared according to the manufacturer’s instruction using between 100 and 1000 ng of total RNA, and the libraries were pooled together and sequenced on the NextSeq 500, 1 × 51.

#### O. bimaculoides and E. scolopes

Small RNA libraries prepared at the Yale University School of Medicine W. M. Keck facility using standard manufacturers’ protocol and sequenced on the Illumina Genome Analyzer II platform loaded onto a single lane.

#### *O. vulgaris* (developmental stages)

The sequencing libraries have been prepared using SMARTer smRNA-Seq kit for Illumina from Clontech according to the manufacturer’s instructions.

#### Quality control and expression quantification

The quality control of the sequencing data has been performed using miRTrace software (v 1.0.1, https://github.com/friedlanderlab/mirtrace) (*60*). MirTrace QC files are available as a part of the Supplementary Materials. MiRDeep2 tool v2.0.1.2 was used to quantify miRNA expression in the tissues.

### miRNA annotation

In general, we followed the annotation procedures described in (*22*). For conserved miRNA discovery, we used MirMachine (https://github.com/sinanugur/MirMachine). Briefly, covariance models for each conserved miRNA family in MirGeneDB (*21*) were searched in the genomes of *O. vulgaris* and *O. bimaculoides*, the bobtail squid *E. scolopes*, and *N. pompilius*. After this, for *Octopus* species and squid (but not the *Nautilus*) species, specific and quality-filtered miRNA sequencing datasets were pooled and used in MirMiner (*61*) for novel miRNA gene discovery in each species. Conserved and novel predictions were compared across cephalopods and other mollusk species in MirGeneDB. Annotation of miRNA families, genes, and paralogs was conducted using synteny and orthology information combined with sequence comparison (with emphasis on seed regions) as described (*21*, *22*).

### miRNA editing analysis

To detect A-to-I editing events in the miRNAs, small RNA sequencing data have been mapped to predicted mature sequences with bowtie aligner allowing maximum two mismatches and no multimapping reads.

Resulting alignment (.bam) files have been processed with a custom Python script to return base counts at every position of the reference. Then, for the reference positions with adenosines having sufficient coverage (more than 10 reads), the proportion of guanosines has been computed. For fig. S7A, the maximum such proportion in miRNA seed (position second-eighth) has been plotted with ComplexHeatmap R package (**62**).

### Alignment of orthologous 3′UTRs

A total of 11,361 one-to-one ortholog pairs between *O. sinensis* and *O. bimaculoides* have been identified using OrthoFinder2 (**63**) with default parameters and proteomes of 17 other representative metazoans (*A. californica*, *B.glabrata*, *B. floridae*, *C. teleta*, *C.intestinalis*, *C. gigas*, *E. scolopes*, *H. sapiens*, *L. anatina*, *L. gigantea*, *M. musculus*, *N. pompilius*, *N. vectensis*, *O. minor*, *P. fucata*, *S. kowalewskii*, and *S. purpuratus*). Then, the coding sequences from *O. sinensis* have been aligned to the genome of *O. bimaculoides* using GMAP (--cross-species, --min-identity = 0.6) (*64*). The alignments have been filtered to include only the cases, where the best alignment for a transcript was the alignment to a corresponding ortholog and where the end of CDS has been aligned precisely to the stop codon of the reference genome. This filtering has resulted in 7,969 alignments. Next, the genomic sequence downstream of the alignment has been extracted. Last, each pair of sequences corresponding to the orthologous 3′UTRs has been aligned with Clustal Omega (**65**) using default parameters.

### *K*-mer conservation scores

The “conservation score” between two *Octopus* species has been then obtained by computing the fraction of cases where a *k*-mer in reference (*O. sinensis*) is matched exactly by the *k*-mer in the query (*O. bimaculoides*) for every distinct *k*-mer. For the analysis, we kept only *k*-mers present at least 10 times in the alignments, and each *k*-mer match has been considered independently (i.e., allowing multiple *k*-mers in the same gene).

For example, Ovu-Let-7 has 77 8-mer MREs detected in 74 genes of *O. vulgaris*. On the basis of coexpression information (we considered miRNA and mRNA “coexpressed” if they have been recovered at 100 and 10 counts per million, respectively, in any of the tissues in the dataset) and conservation, those hits have been divided into four categories:

**Table:** 

	Conservation of an orthologous site in 3′UTR in *O. bimaculoides*	
mRNA:miRNA codetection	Mismatches	Exact match
No codetection	4 sites (4 genes)	5 sites (4 genes)
Codetection	29 sites (27 genes)	39 sites (39 genes)

Then, the conservation score for Ovu-Let-7 would be 39 / (39 + 29) = 0.574 for a subset of cases where mRNA:miRNA pairs are codetected and 5 / (5 + 4) = 0.556 for the cases where they are not (vis data file S3 for counts for all miRNAs). For the Fig. 5B, these values have been converted to the *Z* scores by subtracting the mean and dividing by the standard deviation of conservation values. The list of the all conserved 8-mer sites is available as data file S3. We note that the list greatly underestimates the number of target sites present in the genome because of suboptimal 3′UTR annotation and difficulty of aligning noncoding sequences (i.e., only about 8000 one-to-one ortholog 3′UTRs that could have been aligned out of potentially ~20,000 genes present in either genome). Control 8-mers have been obtained by dinucleotide permutation of MRE 8-mers and selecting the sequences ending in adenosine (to match trailing adenosine in 8-mer MREs).

### One-off MRE conservation

The conservation rates of 8-mers potentially convertible to MREs via a single editing event (one-off MREs) were compared to the conservation rates of control 8-mers with a similar dinucleotide composition obtained by reshuffling as well as 8-mers convertible to MREs via G-to-A substitution.

### Editing of MREs and one-off MREs

To determine whether ADAR is targeting putative MREs and one-off MREs, all such 8-mers conserved between two octopus species have been intersected with A-to-I editing events identified previously. To ensure that the absence of observed editing is not a result of a missing data, only 8-mers with a total RNA-seq coverage of more than 10 mapping reads have been considered.

## Supplementary Materials

### This PDF file includes:

Supplementary Text Figs. S1 to S8 References

## Notes

View/request a protocol for this paper from *Bio-protocol*.
