# Supplementary material for: MicroRNAs are deeply linked to the emergence of the complex octopus brain
Source: Sci Adv. 2022 Nov 25;8(47):eadd9938. doi: 10.1126/sciadv.add9938 (PMC9699675; doi:10.1126/sciadv.add9938)
Supplement: Supplementary file 1 — Supplementary Text Figs. S1 to S8 References [file sciadv.add9938_sm.pdf]

Science Advances
AAAS

Supplementary Materials for

MicroRNAs are deeply linked to the emergence of the complex octopus brain

Grygoriy Zolotarov *et al.*

Corresponding author: Nikolaus Rajewsky, rajewsky@mdc-berlin.de;
Kevin J. Peterson, kevin.j.peterson@dartmouth.edu

Sci. Adv. **8**, eadd9938 (2022)
DOI: 10.1126/sciadv.add9938

The PDF file includes:

Supplementary Text
Figs. S1 to S8
Legends for tables S1 to S5
Legends for data S1 to S4
References

Other Supplementary Material for this manuscript includes the following:

Tables S1 to S5
Data S1 to S4

Supplementary Text

RNA quality assessment

Agilent Bioanalyzer has been used to assess the quality of RNA extracted from the tissues. However, the main quality metric of Bioanalyzer, an RNA integrity Number (RIN), could not be used as it has been developed using mouse and human RNA samples where two ribosomal fragments are expected to dominate total RNA profile. As in the majority of other protostomes (*67*, *68*), 28S RNA in cephalopods bears the so-called "hidden break". Upon denaturation, the 28S rRNA is broken down to two fragments. With an 18S fragment, a total RNA profile of octopus RNA then consists of 3 fragments which makes it impossible for the algorithm to determine RNA quality properly. An example of a profile of intact RNA is shown below (note the disappearance of the 3rd peak upon heat denaturation, the size scale is shifted which is an artifact):

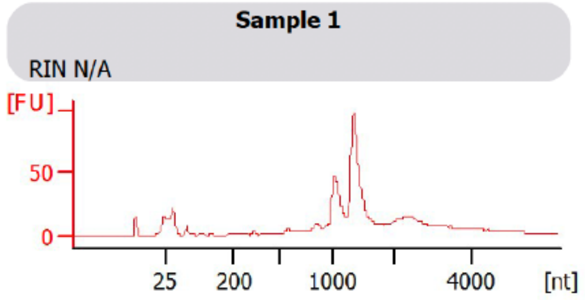

No heat denaturation
hepatopancreas

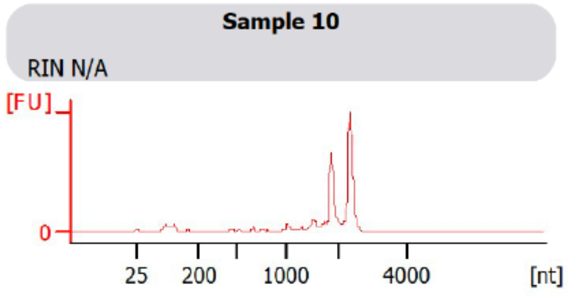

Heat denaturation (70C, 3 min)
hepatopancreas

Nevertheless, degraded RNA can be easily distinguished from intact RNA by observing a region between 5S rRNA and the major peaks. In degraded samples, this region will accumulate irregular peaks corresponding to the degradation fragments or larger ribosomal RNAs. An example for a partially and fully degraded samples are show below:

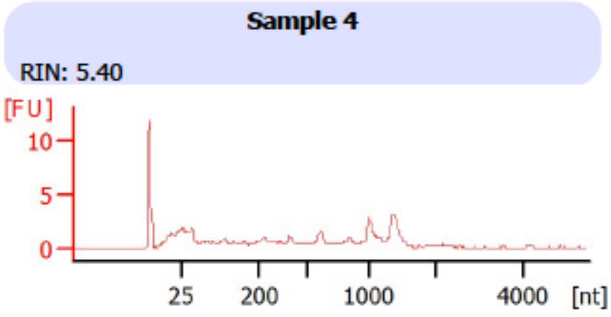

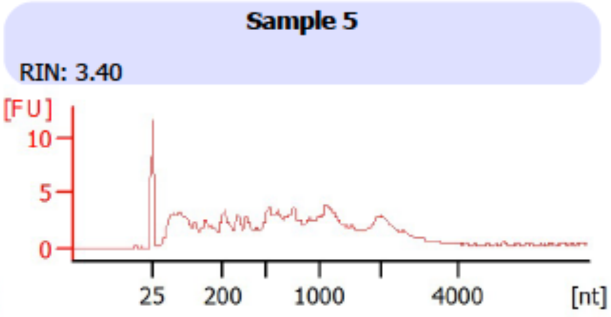

Thus, only samples with clear two or three peaks and no peaks in the intermediate fraction have been selected for the library preparation.

Alternative splicing in the octopus genome

Systematic differences in alternative splicing intensity have been reported between metazoans and unicellular holozoans, early-branching non-bilaterian animals, and bilaterians (*54*) as well as within vertebrate lineage (*55*). In particular, exon-skipping events are widespread among metazoans, with the highest rates observed in bilaterian animals and, in particular, vertebrates. Such increases in the exon-skipping rates have been argued to contribute to an increased phenotypic complexity perhaps by expanding proteome diversity in those lineages (*69*, *70*). An initial assembly and annotation of the *O. bimaculoides* genome suggested no particularly rich alternative splicing of mRNAs (*5*). Genes that are highly alternatively spliced in vertebrates have more isoforms in the octopus genome as well, but the number of such isoforms was lower. In the same study, *O. bimaculoides* has been found to retain 85% of the ancestral introns and the number of novel introns has been found to be comparable to that in other "slow-evolving" spiralians such as *Lottia* and *Capitella*. Here we investigated the extent of alternative splicing by comparing exon skipping and intron retention rates across the tissues of *O. vulgaris* and *O. bimaculoides* (from Albertin et al. 2015 study).

Three million sequencing reads generated by two full-length RNA-seq methods (Iso-seq and FLAM-seq (*16*, *66*)) were used to build a catalog of high-quality mRNAs isoforms (Methods). The isoforms were then filtered (Methods) to produce a final set of 59,579 mRNA isoforms associated with 10,957 reference genes out of 25,335 present in the genome. After filtering, and in combination with a current genome annotation, we estimate that the octopus genome encodes ~80,000 alternative mRNA isoforms. An updated annotation that combines original genome annotation with the isofroms obtained in this study is available as Supplementary Data 1.

We have used our newly assembled transcriptome to characterize alternative splicing in the genome of *O. sinensis* (Methods). *O. sinensis* chromosome-level genome assembly ASM634580v1 (*71*) was used due to its much higher completeness in comparison to a draft genome assembly of *O. vulgaris* (*58*). Both species belong to the same species complex and only recently have been recognized as separate species belonging to the same species complex (*72*, *73*).

In the obtained annotation, 9769 out of 25,335 protein coding genes (38.6%) encoded multiple mRNA isoforms. As in other bilaterians (*54*), the predominant alternative splicing event in the octopus is exon skipping (6513 cases) followed by alternative transcription start and termination. (4773 and 2580 cases respectively), alternative first exon (2313) and intron retention (1927) (Methods). Individual tissues varied in alternative splicing abundance with neuronal tissues often having higher exon-skipping rates (Methods, Fig. S2). We checked whether A-to-I editing may contribute to an increased number of mRNA isoforms by affecting alternative splicing of messenger RNAs. Splicing requires donor and acceptor splicing signals in the mRNA (GT and AG respectively) as well as other splicing-regulatory sequences within introns (branch point, polypyrimidine tract, etc.) As A-to-I editing is thought to occur co-transcriptionally**,** it is feasible for splice sites to be created by adenosine deamination. A notable example of such event in the autoregulatory loop of ADAR2 itself, where adenosine deamination in ADAR2 mRNA creates a proximal splicing acceptor site and results in a premature translation termination due to a frame shift thus possibly acting in self-inhibitory fashion (*74*). However, in human cell lines, ADAR enzymes have been found to target splicing-related motifs only rarely (*75*). If splice donor or/and

acceptor site are created by A-to-I editing, their genomic sequence will be AT or/and AA respectively. To identify such cases, we have mapped available RNA-seq data from representative protostomian species (Methods). As the result, we did not find any non-canonical splice junctions in any of the species investigated (Table S3).

We have also used total RNA-seq data to annotate circular RNAs (circRNAs) in the tissues by detecting back-splicing events (*56*). As ADARs have been shown to antagonize back-splicing events by melting dsRNA structures within introns (*14*), we expected circRNAs in cephalopods to be not as abundant as in other animals. We have predicted circRNAs using total RNA-seq datasets by searching for backsplicing events, and rigorously filtered putative predictions to retain a set of high-quality 296 circRNAs (Methods). These RNAs were expressed in highly tissue-specific manner: the majority (200 / 296) were detected in only one of the tissues, and only 32 in more than 3 tissues.

<u>Alternative cleavage and polyadenylation</u>

We used FLAM-seq to reconstruct mRNA cleavage sites. The sequence context around cleavage sites in octopus strongly resembles that of other metazoans. More precisely, 71% of the sites were canonical metazoan polyadenylation signals (A(A/U)UAAA and variants) (Fig. S3 A-D). Thus, 3'-UTRs and their isoforms are likely generated by a canonical mechanism.

To determine 3'-UTR lengths, we have used FLAMseq data. Genes with higher coverage may theoretically exhibit longer maximal 3'-UTRs as there would be a higher chance of capturing those. However, the relationship between the sequencing depth per gene and the maximal recovered 3'-UTR length is weak and explained about 1% of the variability in the 3'-UTR length (fig. S3E). We thus concluded that our FLAM-seq dataset allows for an unbiased 3'-UTR length estimation for sampled genes. The median length of 3'-UTRs in the octopus was approximately 380 nucleotides. As in other bilaterians, we find that genes expressed in the nervous system utilize, on average, longer 3'UTRs (Fig. S3F) (*76*, *77*)**.** We also found 4,800 genes utilizing tandem alternative polyadenylation sites. As expected, distal cleavage sites usually contained stronger polyadenylation signals (Fig. S3 D) (*78*).

Different genes showed profound differences in 3'-UTR lengths based on whether they have multiple cleavage sites annotated and their tissue expression patterns. Genes with multiple annotated 3'-UTRs exhibited longer 3'-UTRs compared to genes with only one cleavage site captured. The genes specifically expressed in the nervous tissues utilized the longest 3'-UTRs (fig S3). In addition to the lengths of 3'-UTRs, we have investigated steady-state lengths of mRNA poly(A) tails. Similar to the 3'-UTRs, poly(A) tails were longer in the neuronal tissues (123 - 138.5 nt) than in non-neuronal (45-110 nt). The shortest poly(A) tails have been observed in the testis (median length of 45 nt). In addition, mRNA tails in testis showed unusually high percentage of guanosines (up to 10%) (Fig. S3)

Finally, we investigated poly(A) tail lengths. As in other animals, steady-state lengths of poly(A) tails are longer in neuronal tissues (*16*) Interestingly, poly(A) tails in the testis were shorter than in other tissues and contained a high proportion of guanosines (Fig. S3 G,H), which have not been described in any other animal.

Thus, while we found unusual poly(A) tails in the testis, we conclude that the octopus employs polyadenylation patterns similar to those observed in other lophotrochozoans. In summary, the transcriptome of *O. vulgaris* does not show major departures from other lophotrochozoans in terms of alternative splicing diversity and rates, as well as in mRNA cleavage and polyadenylation. This matches a previous observation of gene content and intronic architecture of coleoids resembling that of other "slow-evolving" lophotrochozoans (*5*).

RNA editing index

A-to-I editing index has been computed as in (*59*). The main motivation for using this index instead of defining editing sites is explained below. First of all, it avoids defining RNA editing sites explicitly using an arbitrary RNA sequencing depth threshold. Secondly, different genomic features have different coverage by the RNA sequencing reads. Such uneven coverage leads to more editing sites being defined in the coding sequences per unit of length and may lead to false conclusion that coding sequences are edited at higher levels. Thus, when defining editing sites, the coverage should be accounted for. RNA editing index, on the other hand, is obtained by pulling the information from all reference adenosines for which there is any coverage present. This explicitly accounts for uneven coverage and thus allows comparison of different genomic regions. A hypothetical example illustrating this is shown in (Fig.S1A and B) When explicitly calling editing sites, feature A appears to be edited more (i.e. more editing sites per unit length) than feature B where no editing sites have been called due to low coverage. However, when comparing editing indices, the opposite is true. We have computed the editing index per each type of genomic feature in *O. sinensis* and *O. bimaculoides* genomes using the data generated in this study and (*5*) dataset respectively. Among the genic features in both species, the introns appear to have the highest editing indices followed by 3'-UTRs and coding sequences (Fig. S1).

Dependence between tissue sampling and complement of annotated miRNAs

MiRNAs were annotated in every species *independently* based on genome sequence and small RNA sequencing data (Materials and Methods). In only 4 cases, a putative microRNA locus has lacked reads in one of the species but was then annotated based on sequence similarity of the pre-miRNA in each of the respective genomes (see annotations in Mirgenedb.org):

- Mir-96-P1h (Bilaterian) in *O. vulgaris*
- Mir-10-P6 in (Eumetazoan) *O. bimaculoides*
- Mir-Novel-58 (Octopus) in *O. bimaculoides*
- Mir-Novel-70 (Octopus) in *O. bimaculoides*

Thus, the whole-body dataset for *E. scolopes,* when cross-referenced against the whole-body data set for *O. bimaculoides,* captured the entire known complement of coleoid microRNAs even without the extensive tissue-specific datasets of *O. vulgaris.*

Dependence between sequencing depth and the capture of miRNAs

To understand whether and how sequencing depth would affect miRNA detection rates we have subsampled small-RNA sequencing libraries from all 3 species in the study to the same number of reads (from 100,000 up to 8 millions). For every subsampled dataset, the reads have been aligned to the set of miRNA precursors and the number of detected miRNAs has been recorded (Fig. S5). In *E. scolopes* and *O. bimaculoides*, a sequencing depth of 1M reads is already sufficient to recover all coleoid miRNAs annotated in the genome despite the fact that in the case of *E. scolopes* the sequencing library has been produced from a cell-free hemolymph. In *O. vulgaris*, a sequencing depth of 1-3 million reads has been sufficient to recover all predicted coleoid microRNAs in almost all tissues of the animal. Non-neuronal tissues exhibited higher saturation depths when compared to neuronal tissues (Wilcoxon ranked sum test p-value = 0.0442), while no difference has been found between 2 library preparation methods used in the study (Wilcoxon paired ranked sum test p-value = 0.2).

A-to-I editing of miRNAs

Changes in the seed sequence of a miRNA (positions 2-8 counted from the 5' end) are expected to alter target recognition (*28*). However, similar to mammals (*79*), our data show that miRNA seed regions are not edited to any considerable extent. In the mature miRNA sequences, the mismatches were more abundant towards the 3'-end of the molecule (Fig. S7B). The most common types of mismatches were non-templated 3' additions of cytosine, adenine and uridine (Fig. S7C). When pooling the data from all datasets, the average number of reads mapping to the position in a seed was 49,532 (median 27,967; minimum 12). 146/147 miRNAs have had all 7 bases of the seed profiled with at least 10 sequencing reads and 136 with at least 100 reads. The majority of mismatches were specific to either Truseq (6,443) or Clontech (2,355) datasets and thus probably represent sequencing errors. In the remaining 894 cases, where a particular mismatch has been recovered by both library preparation methods, there was a mild correlation in the estimated mismatch frequencies between the methods (Spearman's rho = 0.46, correlation test p-value < 4.93E-43). In total, 5 miRNAs show signs of editing in their seed sequence above 1%. Of these, only 3 cases have been recovered in multiple tissues and none by both library preparation methods simultaneously (Table S4).

De-novo creation of miRNA targeting sites by RNA editing

Having established the higher conservation for miRNA response elements ("MRE's") compared to non-MRE controls (Main text, Methods), we tallied cases where A-to-I editing possibly created functional MREs by introducing A-to-G substitutions. We reasoned that if such "one-off MREs" (8-mers convertible to an MRE via A-to-G mismatch) exist and are functional, they would, similarly to canonical targeting sites, display higher conservation rates compared to other octamers (vis above). We recorded, genome-wide, the conservation of such one-off MREs and observed no trace of higher conservation when compared to G-to-A one-off controls (i.e. octamers convertible to an MRE via G-to-A substitution, Fig. 8E, p > 0.05, Wilcoxon rank sum test with continuity correction). Furthermore, we have checked whether one-off MREs with editing events had higher conservation rates compared to the same type of 8-mers not targeted by ADAR and found no difference. Out of 71,772 one-off MREs conserved between 2 octopus species (306 sequences), only 159 (57 different sequences) have been found to be targeted by

ADAR. These 57 one-off MREs were conserved at the same rates as their unedited counterparts ($p > 0.05$; Wilcoxon rank sum test). This suggests that the de-novo creation of miRNA targeting sites via A-to-I editing may not be a widespread phenomenon in cephalopods. Similarly, A-to-I editing events with the potential to destroy the target sites have been found to happen rarely. Out of 10,053 MREs conserved between 2 octopus species, only 39 (0.3%) have been found to be targeted by ADAR (Methods). This is in line with the known preference of ADAR for the double-stranded RNA and the general depletion of the secondary RNA structure around functional miRNA targeting sites (*80*).

Supplementary Figures

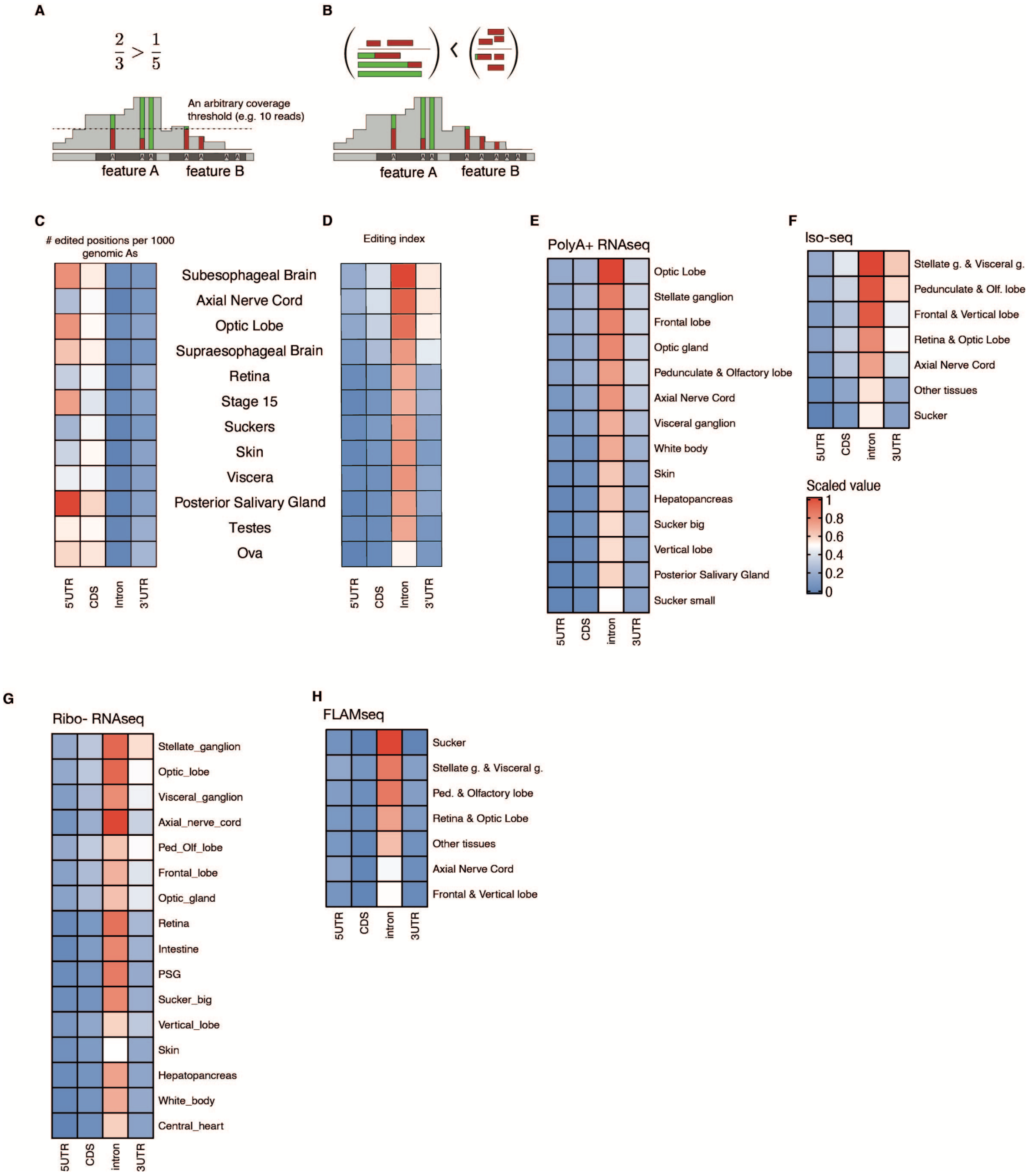
A
2/3 > 1/5
An arbitrary coverage threshold (e.g. 10 reads)
feature A
feature B
B
feature A
feature B
C
edited positions per 1000 genomic As
Subesophageal Brain
Axial Nerve Cord
Optic Lobe
Supraesophageal Brain
Retina
Stage 15
Suckers
Skin
Viscera
Posterior Salivary Gland
Testes
Ova
5'UTR
CDS
Intron
3'UTR
D
Editing index
5'UTR
CDS
Intron
3'UTR
E
PolyA+ RNAseq
Optic Lobe
Stellate ganglion
Frontal lobe
Optic gland
Pedunculate & Olfactory lobe
Axial Nerve Cord
Visceral ganglion
White body
Skin
Hepatopancreas
Sucker big
Vertical lobe
Posterior Salivary Gland
Sucker small
5UTR
CDS
intron
3UTR
F
Iso-seq
Stellate g. & Visceral g.
Pedunculate & Olf. lobe
Frontal & Vertical lobe
Retina & Optic Lobe
Axial Nerve Cord
Other tissues
Sucker
5UTR
CDS
intron
3UTR
Scaled value
1
0.8
0.6
0.4
0.2
0
G
Ribo- RNAseq
Stellate_ganglion
Optic_lobe
Visceral_ganglion
Axial_nerve_cord
Ped_Olf_lobe
Frontal_lobe
Optic_gland
Retina
Intestine
PSG
Sucker_big
Vertical_lobe
Skin
Hepatopancreas
White_body
Central_heart
5UTR
CDS
intron
3UTR
H
FLAMseq
Sucker
Stellate g. & Visceral g.
Ped. & Olfactory lobe
Retina & Optic Lobe
Other tissues
Axial Nerve Cord
Frontal & Vertical lobe
5UTR
CDS
intron
3UTR

Fig. S1. Genomic targets of A-to-I editing

(A) Definition of calling editing sites. Editing sites are required to have minimal coverage (e.g., 10 RNA-seq reads) to increase the precision. Some genomic features (e.g., introns) will not have coverage above the threshold across their whole length. Thus, normalization by feature-length will lead to the underestimation of editing intensity in such loci. **(B)** Editing index approach introduced in [76]. A-to-G mismatches are pooled across the whole feature. Note that the index implicitly accounts for the coverage and uncovered positions have no effect on the resulting value. **(C** and **D)** An average number of called editing sites (C) or editing index (D) per type of genomic feature in each tissue of *O. bimaculoides* (dataf from [5]). The values have been normalized such that the maximal and minimal value per matrix is 1 and 0 respectively. While neuronal tissues show higher values in both measures, the most edited features differ. **(E-G)** Average editing indices of genomic features of *O. vulgaris* for every RNA-sequencing dataset generated in this study.

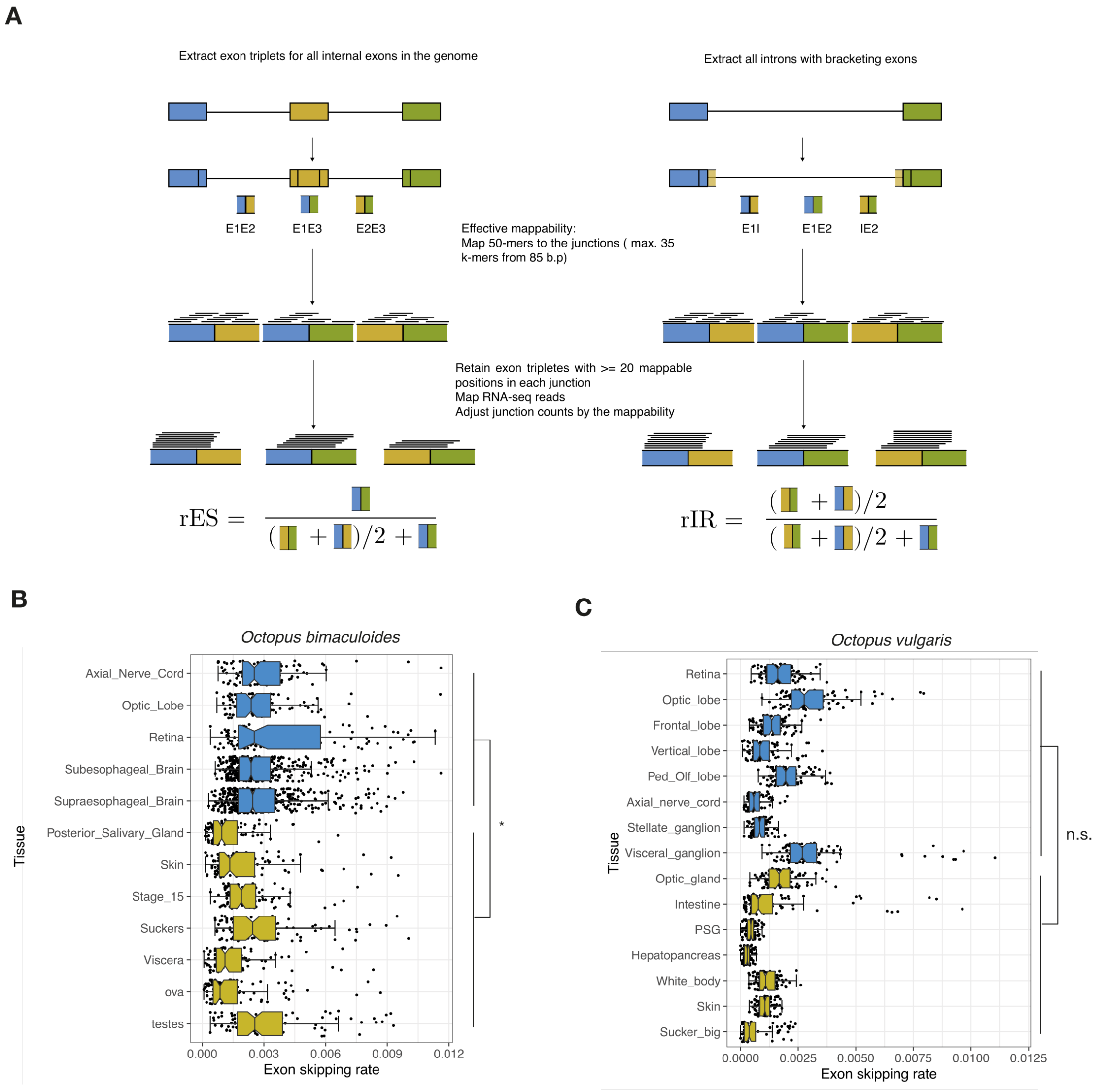
A
Extract exon triplets for all internal exons in the genome
Extract all introns with bracketing exons
E1E2
E1E3
E2E3
Effective mappability:
Map 50-mers to the junctions (max. 35 k-mers from 85 b.p)
E1I
E1E2
IE2
Retain exon tripletes with >= 20 mappable positions in each junction
Map RNA-seq reads
Adjust junction counts by the mappability
rES =
rIR =
B
Octopus bimaculoides
Axial_Nerve_Cord
Optic_Lobe
Retina
Subesophageal_Brain
Supraesophageal_Brain
Posterior_Salivary_Gland
Skin
Stage_15
Suckers
Viscera
ova
testes
Tissue
0.000
0.003
0.006
0.009
0.012
Exon skipping rate
*
C
Octopus vulgaris
Retina
Optic_lobe
Frontal_lobe
Vertical_lobe
Ped_Olf_lobe
Axial_nerve_cord
Stellate_ganglion
Visceral_ganglion
Optic_gland
Intestine
PSG
Hepatopancreas
White_body
Skin
Sucker_big
Tissue
0.0000
0.0025
0.0050
0.0075
0.0100
0.0125
Exon skipping rate
n.s.

Fig. S2. Alternative splicing rates across tissues

(A) General approach used for quantification exon skipping and intron retention rates; adapted from (*54*). **(B** and **C)** Exon skipping rates in representative tissues of *O. bimaculoides* (**B**, data from (*5*)) and *O. vulgaris* (**C**) (Methods). Wilcoxon rank sum test with continuity correction was used to test for the higher median per-tissue exon skipping rate between neuronal and non-neuronal tissue types, * $p< 0.05$,** $p < 0.01$, *** $p < 0.001$

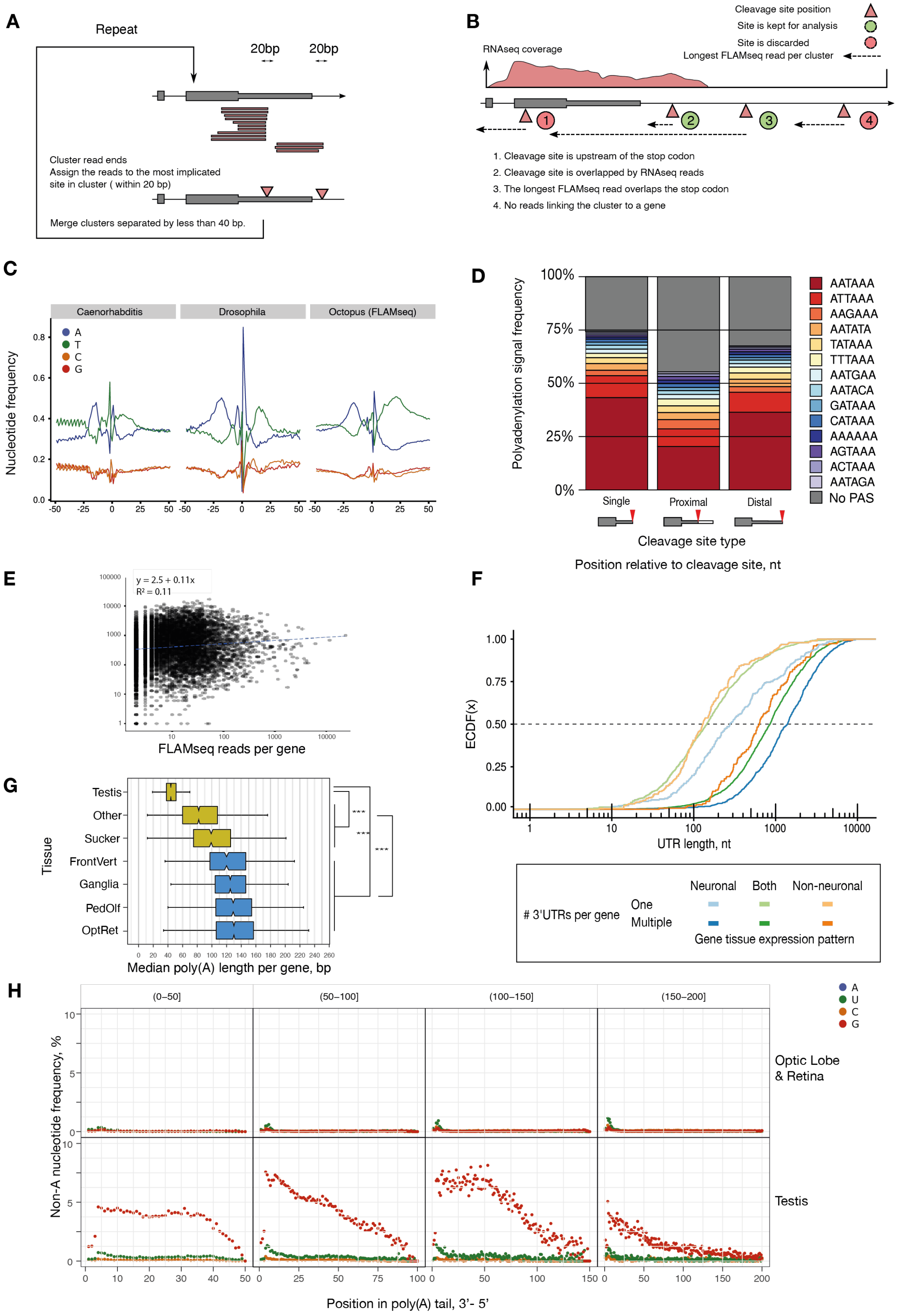
A
Repeat
20bp
20bp
Cluster read ends
Assign the reads to the most implicated site in cluster (within 20 bp)
Merge clusters separated by less than 40 bp.
B
Cleavage site position
Site is kept for analysis
Site is discarded
Longest FLAMseq read per cluster
RNAseq coverage
1. Cleavage site is upstream of the stop codon
2. Cleavage site is overlapped by RNAseq reads
3. The longest FLAMseq read overlaps the stop codon
4. No reads linking the cluster to a gene
C
Caenorhabditis
Drosophila
Octopus (FLAMseq)
Nucleotide frequency
Position relative to cleavage site, nt
D
Polyadenylation signal frequency
Single
Proximal
Distal
Cleavage site type
AATAAA
ATTAAA
AAGAAA
AATATA
TATAAA
TTTAAA
AATGAA
AATACA
GATAAA
CATAAA
AAAAAA
AGTAAA
ACTAAA
AATAGA
No PAS
E
y = 2.5 + 0.11x
R² = 0.11
FLAMseq reads per gene
F
ECDF(x)
UTR length, nt
3'UTRs per gene
One
Multiple
Neuronal
Both
Non-neuronal
Gene tissue expression pattern
G
Tissue
Testis
Other
Sucker
FrontVert
Ganglia
PedOlf
OptRet
Median poly(A) length per gene, bp
H
(0–50]
(50–100]
(100–150]
(150–200]
Optic Lobe & Retina
Testis
Non-A nucleotide frequency, %
Position in poly(A) tail, 3'- 5'

Fig. S3. mRNA cleavage and polyadenylation

(A) A general approach of assigning FLAM-seq tags to genes. **(B)** A hypothetical locus with examples of putative mRNA cleavage sites with various supporting evidence and different results of filtering. **(C)** Sequence profiles around cleavage sites in representative protostomian species. **(D)** Proportions of polyadenylation signals identified within 40 b.p upstream of the cleavage sites. **(E)** Relationship between FLAMseq coverage and estimated 3'-UTR length per gene. Blue line and the formula describe an estimated linear relationship between 2 variables. **(F)** Empirical cumulative distribution function of 3'-UTR length for various groups of genes in the *O. sinensis* genome. **(G)** Median mRNA poly(A) tail lengths per gene in different tissues as determined by FLAMseq. Only genes with at least 5 distinct UMIs are shown. (Supplementary Text). Wilcoxon rank sum test with continuity correction, * $p< 0.05$,** $p < 0.01$, *** $p < 0.001$ **(H)** Non-adenosine nucleotide proportions across mRNA tails in testis and neuronal tissue (optic lobe and retina). The tails have been grouped into 4 length bins and aligned such that 0 refers to the 3'-most base.

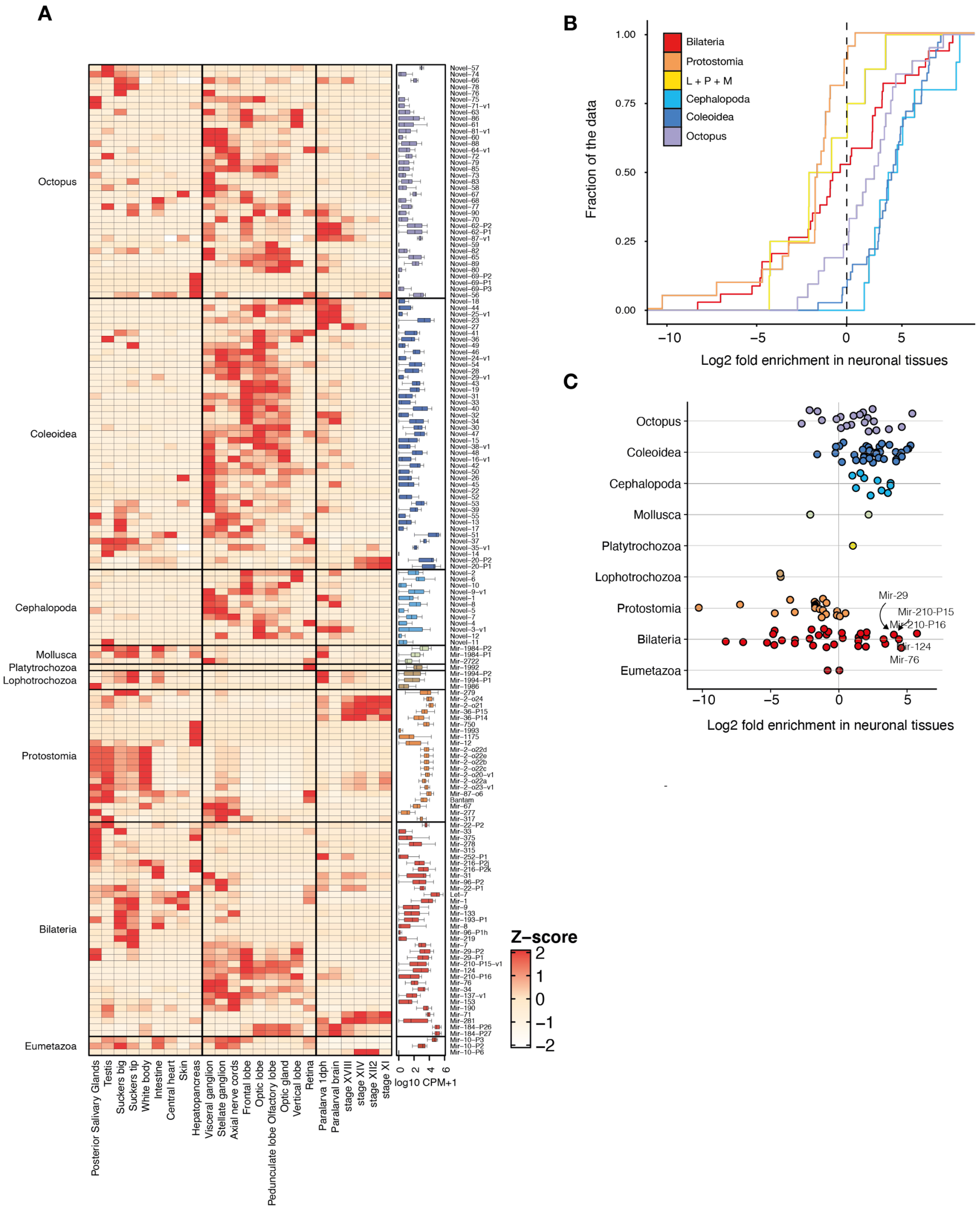
A
Octopus
Coleoidea
Cephalopoda
Mollusca
Platytrochozoa
Lophotrochozoa
Protostomia
Bilateria
Eumetazoa
Posterior Salivary Glands
Testis
Suckers big
Suckers tip
White body
Intestine
Central heart
Skin
Hepatopancreas
Visceral ganglion
Stellate ganglion
Axial nerve cords
Frontal lobe
Optic lobe
Peduncluate lobe Olfactory lobe
Optic gland
Vertical lobe
Retina
Paralarva 1dph
Paralarval brain
stage XVIII
stage XIV
stage XII2
stage XI
0 2 4 6
log10 CPM+1
Z-score
2
1
0
−1
−2
B
Bilateria
Protostomia
L + P + M
Cephalopoda
Coleoidea
Octopus
Fraction of the data
1.00
0.75
0.50
0.25
0.00
−10
−5
0
5
Log2 fold enrichment in neuronal tissues
C
Octopus
Coleoidea
Cephalopoda
Mollusca
Platytrochozoa
Lophotrochozoa
Protostomia
Bilateria
Eumetazoa
Mir-29
Mir-210-P15
Mir-210-P16
Mir-124
Mir-76
−10
−5
0
5
Log2 fold enrichment in neuronal tissues

Fig. S4. Extended expression patterns and neuronal enrichment of miRNAs

(A) Detailed miRNA expression heatmap as in Fig.1. Boxplots on the right summarize distributions of counts-per-million (CPMs) across tissues for every miRNA. **(B** and **C)** Log2 fold enrichment in neuronal tissues. Only miRNAs detected with at least 3 CPMs in both neuronal and non-neuronal tissues are shown. Enrichment is defined as the ratio of average expression values in neuronal and non-neuronal tissue types. In (**B)**, miRNAs with origins in Lophotrochozoa, Platytrochozoa, and Mollusca lineages have been grouped together (L + P + M) due to a low number of genes in individual groups; for the same reason, miRNAs of Eumetazoan origin have been omitted. In **C**, top 5 of the bilaterian miRNAs with the highest neuronal enrichment are labelled.

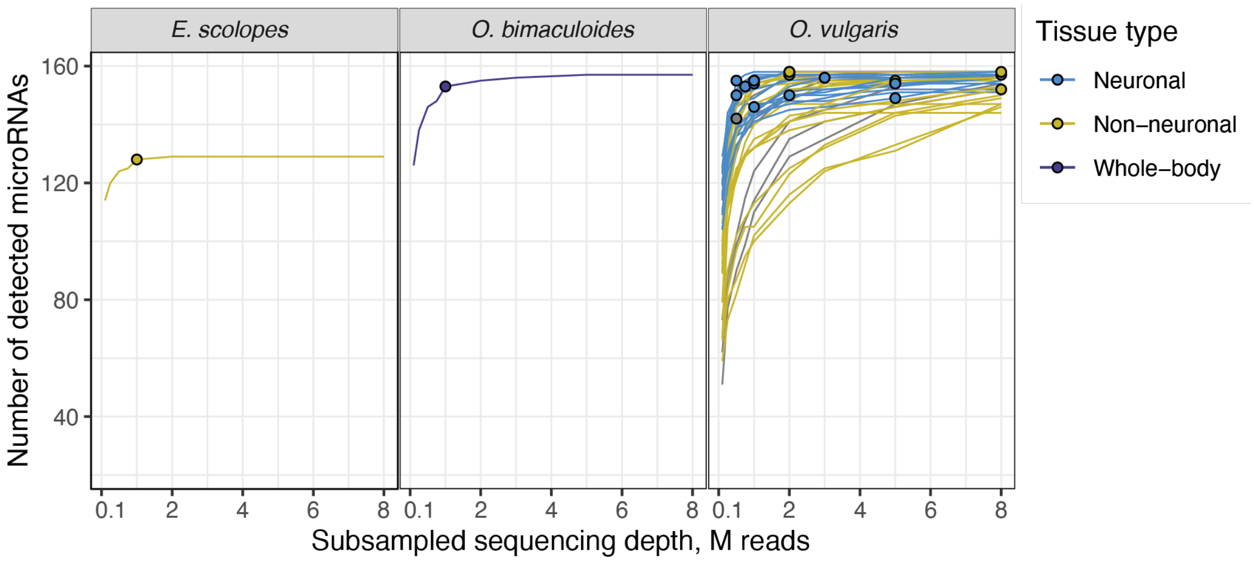

Fig. S5. Sequencing depth and miRNA capture

Relationship between subsampled sequencing depth (x-axis) per library and the number of captured miRNAs for every species and small RNA sequencing library used in the study. For each library, a total number of miRNA precursor sequences with at least one mapping read has been counted. Dots specify the depth at which *all* coleoid miRNAs have been captured (if such saturation has been achieved). For further details, vis Supplementary Text.

.

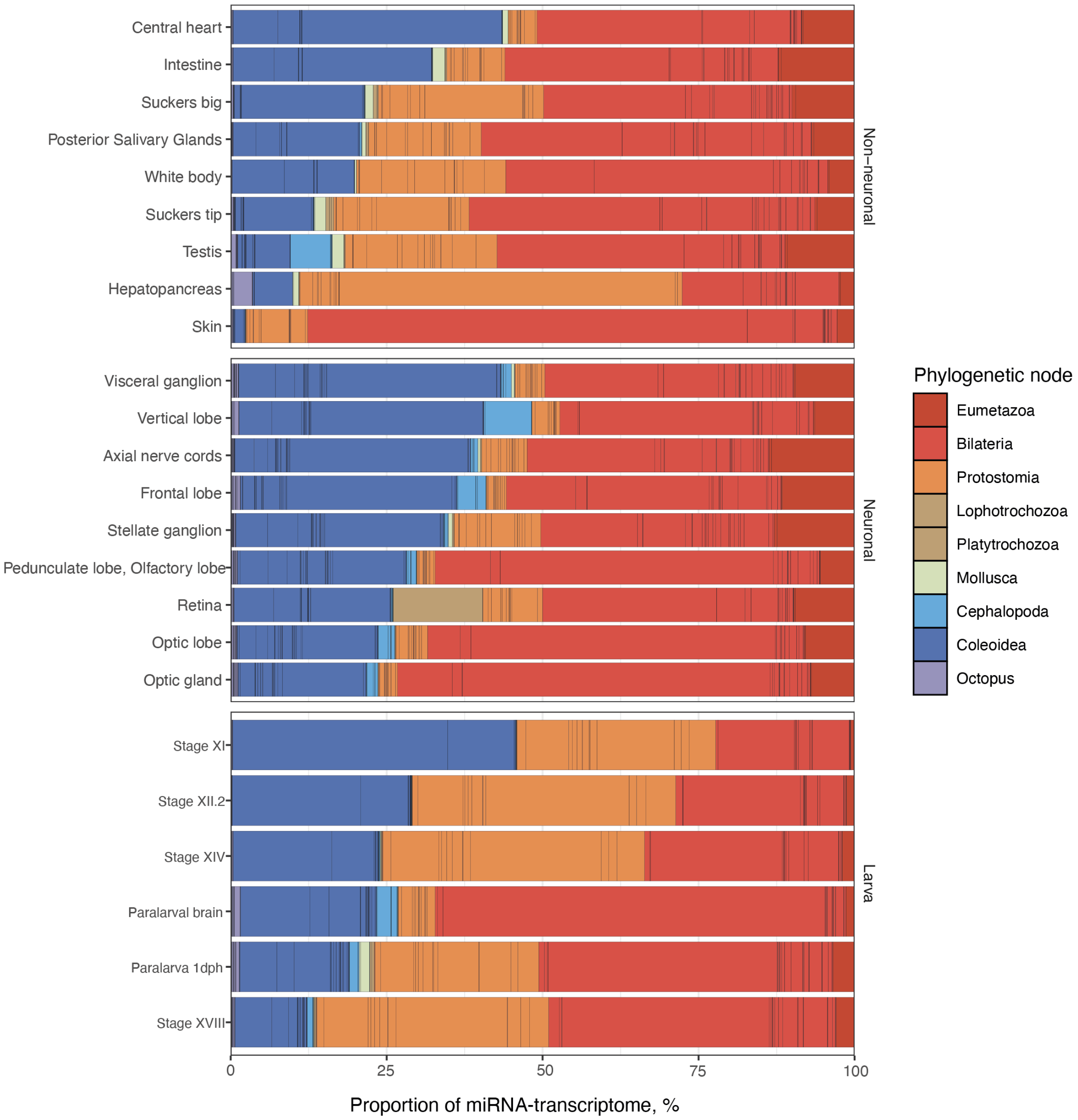

Fig. S6. Fraction of transcriptome dedicated miRNAs of different phylogenetic ages

MiRNA expression as fraction of counts (as quantified by miRDeep2) colored according to the phylogenetic node of origin in all tissues profiled in the study. Tissues in have been sorted according to the proportion of reads assigned to miRNAs of coleoid origins.

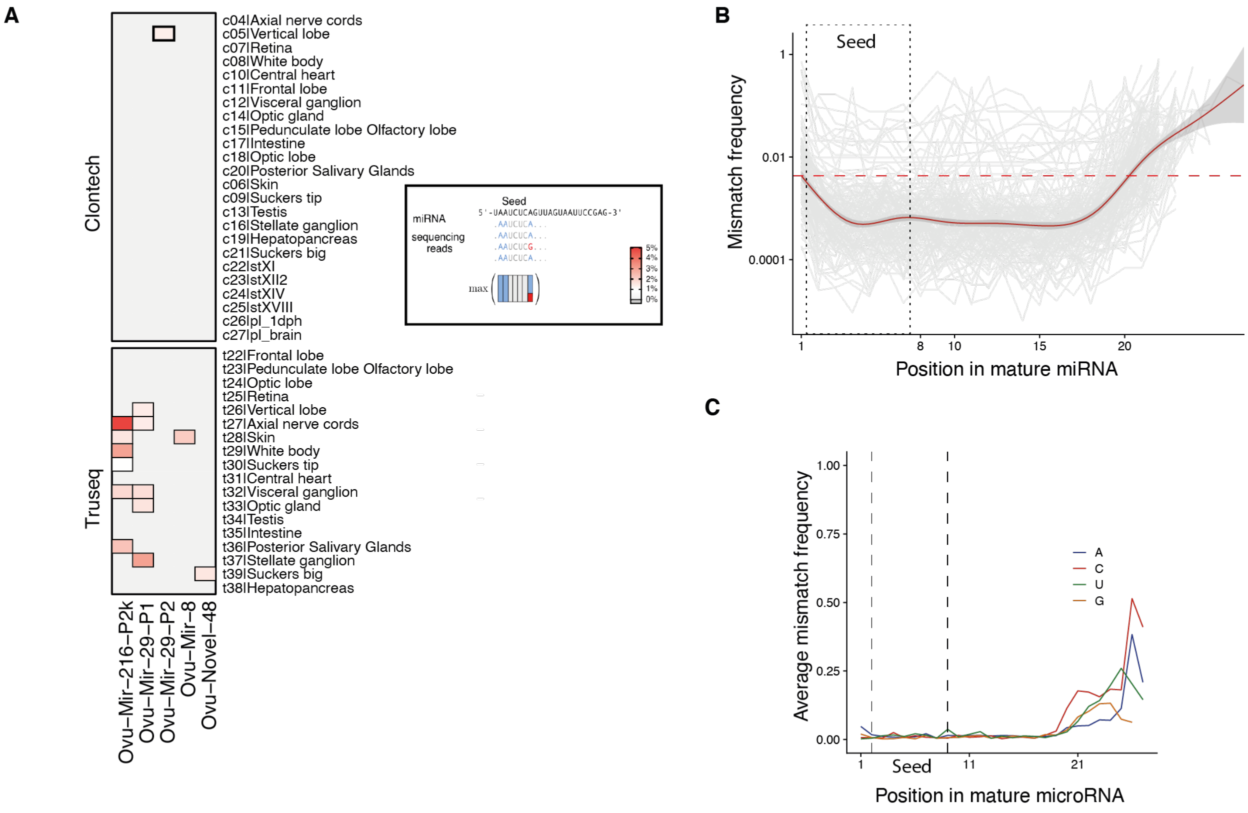

Fig. S7 Editing of miRNAs

(A). Heatmap of maximal editing levels captured within miRNA seeds for all 5 miRNAs with editing levels above 1% in at least one of the tissues.

(B). Total proportion of mismatches recovered for every position in individual miRNAs (gray lines). The horizontal red line marks a median sequencing error rate for Illumina NextSeq machine used in the study (0.429% from (*81*)).

(C). Total proportion of mismatches called at different positions of miRNAs separated by the alternative base type.

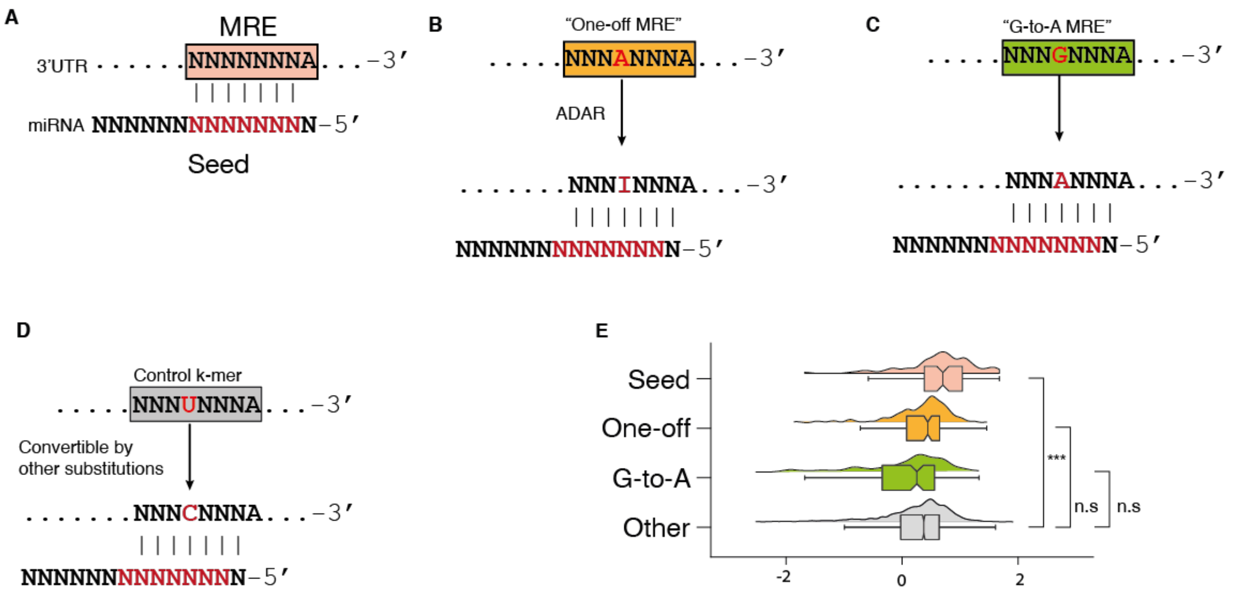

Fig. S8 Conservation of 8-mers potentially convertible to MRE by A-to-I editing

(A to **D)** Diagrams illustrating the types of k-mers compared in the panel **(E)**. "One-off MRE" – an 8-mer potentially convertible to MRE via A-to-I substitution; "G-to-A MRE" – an 8-mer convertible to MRE by a single G-to-A substitution; "Other" – 8-mers differing by 1 base from MREs and thus convertible to MRE via a single substitution that is not A-to-I or G-to-A. **(E)** Conservation Z-scores for the 8-mers (Supplementary Text; Wilcoxon rank sum test with continuity correction, * $p< 0.05$,** $p < 0.01$, *** $p < 0.001$)

Supplementary Data

Data S1. (separate file)

Octopus sinensis genome annotation generated in this study

Data S2. (separate file)

Conservation proportions of microRNA response elements in the 3'-UTR alignments between *O. sinensis* and *O. bimaculoides*

Data S3. (separate file)

A list of putative 8-mer and 7-mer microRNA response elements conserved between *O. sinensis* and *O. bimaculoides*

Data S4. (separate file)

MiRTrace quality control files for small RNA-seq libraries generated in this study

Supplementary Tables

Table S1. (separate file)

Overview of datasets generated in this study (*O. vulgaris* only)

Table S2. (separate file)

Complement of transcription factors in *O. vulgaris* genome

Table S3. (separate file)

Alternative splicing in *O. vulgaris*

Table S4. (separate file)

Maximal detected microRNA editing levels

Table S5. (separate file)

Conserved expression patterns of bilaterian microRNAs

REFERENCES AND NOTES

1. B. Hochner, T. Shomrat, G. Fiorito, The octopus: A model for a comparative analysis of the evolution of learning and memory mechanisms. *Biol. Bull.* **210**, 308–317 (2006).

2. J. Z. Young, Anatomy of the nervous system of *Octopus vulgaris* (1971); https://agris.fao.org/agris-search/search.do?recordID=US201300479148.

3. S. Shigeno, P. L. R. Andrews, G. Ponte, G. Fiorito, Cephalopod brains: An overview of current knowledge to facilitate comparison with vertebrates. *Front. Physiol.* **9**, 952 (2018).

4. W.-S. Chung, N. D. Kurniawan, N. J. Marshall, Comparative brain structure and visual processing in octopus from different habitats. *Curr. Biol.* **32**, 97–110.e4 (2022).

5. C. B. Albertin, O. Simakov, T. Mitros, Z. Y. Wang, J. R. Pungor, E. Edsinger-Gonzales, S. Brenner, C. W. Ragsdale, D. S. Rokhsar, The octopus genome and the evolution of cephalopod neural and morphological novelties. *Nature* **524**, 220–224 (2015).

6. M. Belcaid, G. Casaburi, S. J. McAnulty, H. Schmidbaur, A. M. Suria, S. Moriano-Gutierrez, M. S. Pankey, T. H. Oakley, N. Kremer, E. J. Koch, A. J. Collins, H. Nguyen, S. Lek, I. Goncharenko-Foster, P. Minx, E. Sodergren, G. Weinstock, D. S. Rokhsar, M. McFall-Ngai, O. Simakov, J. S. Foster, S. V. Nyholm, Symbiotic organs shaped by distinct modes of genome evolution in cephalopods. *Proc. Natl. Acad. Sci. U.S.A.* **116**, 3030–3035 (2019).

7. O. Simakov, F. Marletaz, S.-J. Cho, E. Edsinger-Gonzales, P. Havlak, U. Hellsten, D.-H. Kuo, T. Larsson, J. Lv, D. Arendt, R. Savage, K. Osoegawa, P. de Jong, J. Grimwood, J. A. Chapman, H. Shapiro, A. Aerts, R. P. Otillar, A. Y. Terry, J. L. Boore, I. V. Grigoriev, D. R. Lindberg, E. C. Seaver, D. A. Weisblat, N. H. Putnam, D. S. Rokhsar, Insights into bilaterian evolution from three spiralian genomes. *Nature* **493**, 526–531 (2013).

8. S. Alon, S. C. Garrett, E. Y. Levanon, S. Olson, B. R. Graveley, J. J. C. Rosenthal, E. Eisenberg, The majority of transcripts in the squid nervous system are extensively recoded by A-to-I RNA editing. *eLife* **4**, e05198 (2015).

9. N. Liscovitch-Brauer, S. Alon, H. T. Porath, B. Elstein, R. Unger, T. Ziv, A. Admon, E. Y. Levanon, J. J. C. Rosenthal, E. Eisenberg, Trade-off between transcriptome plasticity and genome evolution in cephalopods. *Cell* **169**, 191–202.e11 (2017).

10. L. F. Grice, B. M. Degnan, The origin of the ADAR gene family and animal RNA editing. *BMC Evol. Biol.* **15**, 4 (2015).

11. D. Jiang, J. Zhang, The preponderance of nonsynonymous A-to-I RNA editing in coleoids is nonadaptive. *Nat. Commun.* **10**, 5411 (2019).

12. Y. Shoshan, N. Liscovitch-Brauer, J. J. C. Rosenthal, E. Eisenberg, Adaptive proteome diversification by nonsynonymous A-to-I RNA editing in coleoid cephalopods. *Mol. Biol. Evol.* **38**, 3775–3788 (2021).

13. Y. A. Savva, J. E. C. Jepson, Y.-J. Chang, R. Whitaker, B. C. Jones, G. St Laurent, M. R. Tackett, P. Kapranov, N. Jiang, G. Du, S. L. Helfand, R. A. Reenan, RNA editing regulates transposon-mediated heterochromatic gene silencing. *Nat. Commun.* **4**, 2745 (2013).

14. A. Ivanov, S. Memczak, E. Wyler, F. Torti, H. T. Porath, M. R. Orejuela, M. Piechotta, E. Y. Levanon, M. Landthaler, C. Dieterich, N. Rajewsky, Analysis of intron sequences reveals hallmarks of circular RNA biogenesis in animals. *Cell Rep.* **10**, 170–177 (2015).

15. M. Pujantell, E. Riveira-Muñoz, R. Badia, M. Castellví, E. Garcia-Vidal, G. Sirera, T. Puig, C. Ramirez, B. Clotet, J. A. Esté, E. Ballana, RNA editing by ADAR1 regulates innate and antiviral immune functions in primary macrophages. *Sci. Rep.* **7**, 13339 (2017).

16. I. Legnini, J. Alles, N. Karaiskos, S. Ayoub, N. Rajewsky, FLAM-seq: Full-length mRNA sequencing reveals principles of poly(A) tail length control. *Nat. Methods* **16**, 879–886 (2019).

17. A. Rybak-Wolf, C. Stottmeister, P. Glažar, M. Jens, N. Pino, S. Giusti, M. Hanan, M. Behm, O. Bartok, R. Ashwal-Fluss, M. Herzog, L. Schreyer, P. Papavasileiou, A. Ivanov, M. Öhman, D. Refojo, S. Kadener, N. Rajewsky, Circular RNAs in the mammalian brain are highly abundant, conserved, and dynamically expressed. *Mol. Cell* **58**, 870–885 (2015).

18. K. Chen, N. Rajewsky, Deep conservation of microRNA-target relationships and 3’UTR motifs in vertebrates, flies, and nematodes. *Cold Spring Harb. Symp. Quant. Biol.* **71**, 149–156 (2006).

19. M. R. Friedländer, W. Chen, C. Adamidi, J. Maaskola, R. Einspanier, S. Knespel, N. Rajewsky, Discovering microRNAs from deep sequencing data using miRDeep. *Nat. Biotechnol.* **26**, 407–415 (2008).

20. A. R. Tanner, D. Fuchs, I. E. Winkelmann, M. T. P. Gilbert, M. S. Pankey, Â. M. Ribeiro, K. M. Kocot, K. M. Halanych, T. H. Oakley, R. R. da Fonseca, D. Pisani, J. Vinther, Molecular clocks indicate turnover and diversification of modern coleoid cephalopods during the Mesozoic Marine Revolution. *Proc. Biol. Sci.* **284**, 20162818 (2017).

21. B. Fromm, E. Høye, D. Domanska, X. Zhong, E. Aparicio-Puerta, V. Ovchinnikov, S. U. Umu, P. J. Chabot, W. Kang, M. Aslanzadeh, M. Tarbier, E. Mármol-Sánchez, G. Urgese, M. Johansen, E. Hovig, M. Hackenberg, M. R. Friedländer, K. J. Peterson, MirGeneDB 2.1: Toward a complete sampling of all major animal phyla. *Nucleic Acids Res.* **50**, D204–D210 (2021).

22. B. Fromm, T. Billipp, L. E. Peck, M. Johansen, J. E. Tarver, B. L. King, J. M. Newcomb, L. F. Sempere, K. Flatmark, E. Hovig, K. J. Peterson, A uniform system for the annotation of vertebrate microRNA genes and the evolution of the human microRNAome. *Annu. Rev. Genet.* **49**, 213–242 (2015).

23. Y. Zhang, F. Mao, H. Mu, M. Huang, Y. Bao, L. Wang, N.-K. Wong, S. Xiao, H. Dai, Z. Xiang, M. Ma, Y. Xiong, Z. Zhang, L. Zhang, X. Song, F. Wang, X. Mu, J. Li, H. Ma, Y. Zhang, H. Zheng, O. Simakov, Z. Yu, The genome of *Nautilus pompilius* illuminates eye evolution and biomineralization. *Nat. Ecol. Evol.* **5**, 927–938 (2021).

24. S. Lianoglou, V. Garg, J. L. Yang, C. S. Leslie, C. Mayr, Ubiquitously transcribed genes use alternative polyadenylation to achieve tissue-specific expression. *Genes Dev.* **27**, 2380–2396 (2013).

25. F. Christodoulou, F. Raible, R. Tomer, O. Simakov, K. Trachana, S. Klaus, H. Snyman, G. J. Hannon, P. Bork, D. Arendt, Ancient animal microRNAs and the evolution of tissue identity. *Nature* **463**, 1084–1088 (2010).

26. H. Iwama, K. Kato, H. Imachi, K. Murao, T. Masaki, Human microRNAs originated from two periods at accelerated rates in mammalian evolution. *Mol. Biol. Evol.* **30**, 613–626 (2013).

27. Y. Zhao, G.-A. Lu, H. Yang, P. Lin, Z. Liufu, T. Tang, J. Xu, Run or die in the evolution of new MicroRNAs-testing the Red Queen hypothesis on de novo new genes. *Mol. Biol. Evol.* **38**, 1544–1553 (2021).

28. B. P. Lewis, C. B. Burge, D. P. Bartel, Conserved seed pairing, often flanked by adenosines, indicates that thousands of human genes are microRNA targets. *Cell* **120**, 15–20 (2005).

29. A. Krek, D. Grün, M. N. Poy, R. Wolf, L. Rosenberg, E. J. Epstein, P. MacMenamin, I. da Piedade, K. C. Gunsalus, M. Stoffel, N. Rajewsky, Combinatorial microRNA target predictions. *Nat. Genet.* **37**, 495–500 (2005).

30. P. Sood, A. Krek, M. Zavolan, G. Macino, N. Rajewsky, Cell-type-specific signatures of microRNAs on target mRNA expression. *Proc. Natl. Acad. Sci. U.S.A.* **103**, 2746–2751 (2006).

31. K. Chen, N. Rajewsky, The evolution of gene regulation by transcription factors and microRNAs. *Nat. Rev. Genet.* **8**, 93–103 (2007).

32. B. Deline, J. M. Greenwood, J. W. Clark, M. N. Puttick, K. J. Peterson, P. C. J. Donoghue, Evolution of metazoan morphological disparity. *Proc. Natl. Acad. Sci. U.S.A.* **115**, E8909–E8918 (2018).

33. J. W. Valentine, A. G. Collins, C. P. Meyer, Morphological complexity increase in Metazoans. *Paleobiology* **20**, 131–142 (1994).

34. C. B. Albertin, S. Medina-Ruiz, T. Mitros, H. Schmidbaur, G. Sanchez, Z. Y. Wang, J. Grimwood, J. J. C. Rosenthal, C. W. Ragsdale, O. Simakov, D. S. Rokhsar, Genome and transcriptome mechanisms driving cephalopod evolution. *Nat. Commun.* **13**, 2427 (2022).

35. S. Garrett, J. J. C. Rosenthal, RNA editing underlies temperature adaptation in K+ channels from polar octopuses. *Science* **335**, 848–851 (2012).

36. B. Wu, X. Chen, M. Yu, J. Ren, J. Hu, C. Shao, L. Zhou, X. Sun, T. Yu, Y. Zheng, Y. Wang, Z. Wang, H. Zhang, G. Fan, Z. Liu, Chromosome-level genome and population genomic analysis provide insights into the evolution and environmental adaptation of Jinjiang oyster *Crassostrea ariakensis*. *Mol. Ecol. Resour.* **22**, 1529–1544 (2022).

37. H. Schmidbaur, A. Kawaguchi, T. Clarence, X. Fu, O. P. Hoang, B. Zimmermann, E. A. Ritschard, A. Weissenbacher, J. S. Foster, S. V. Nyholm, P. A. Bates, C. B. Albertin, E. Tanaka, O. Simakov, Emergence of novel cephalopod gene regulation and expression through large-scale genome reorganization. *Nat. Commun.* **13**, 2172 (2022).

38. A. M. Heimberg, L. F. Sempere, V. N. Moy, P. C. J. Donoghue, K. J. Peterson, MicroRNAs and the advent of vertebrate morphological complexity. *Proc. Natl. Acad. Sci. U.S.A.* **105**, 2946–2950 (2008).

39. X. Li, J. J. Cassidy, C. A. Reinke, S. Fischboeck, R. W. Carthew, A microRNA imparts robustness against environmental fluctuation during development. *Cell* **137**, 273–282 (2009).

40. M. S. Ebert, P. A. Sharp, Roles for microRNAs in conferring robustness to biological processes. *Cell* **149**, 515–524 (2012).

41. J. J. Cassidy, A. R. Jha, D. M. Posadas, R. Giri, K. J. T. Venken, J. Ji, H. Jiang, H. J. Bellen, K. P. White, R. W. Carthew, miR-9a minimizes the phenotypic impact of genomic diversity by buffering a transcription factor. *Cell* **155**, 1556–1567 (2013).

42. J. M. Schmiedel, S. L. Klemm, Y. Zheng, A. Sahay, N. Blüthgen, D. S. Marks, A. van Oudenaarden, MicroRNA control of protein expression noise. *Science* **348**, 128–132 (2015).

43. E. Hornstein, N. Shomron, Canalization of development by microRNAs. *Nat. Genet.* **38** Suppl, S20–S24 (2006).

44. K. J. Peterson, M. R. Dietrich, M. A. McPeek, MicroRNAs and metazoan macroevolution: Insights into canalization, complexity, and the Cambrian explosion. *Bioessays* **31**, 736–747 (2009).

45. C.-I. Wu, Y. Shen, T. Tang, Evolution under canalization and the dual roles of microRNAs—A hypothesis. *Genome Res.* **19**, 734–743 (2009).

46. R. Styfhals, G. Zolotarov, G. Hulselmans, K. I. Spanier, S. Poovathingal, A. M. Elagoz, A. Deryckere, N. Rajewsky, G. Ponte, G. Fiorito, S. Aerts, E. Seuntjens, Cell type diversity in a developing octopus brain. bioRxiv 2022.01.24.477459 [**Preprint**]. 24 January 2022. https://doi.org/10.1101/2022.01.24.477459.

47. L. F. Sempere, C. N. Cole, M. A. McPeek, K. J. Peterson, The phylogenetic distribution of metazoan microRNAs: Insights into evolutionary complexity and constraint. *J. Exp. Zool. B Mol. Dev. Evol.* **306**, 575–588 (2006).

48. G. M. Schratt, F. Tuebing, E. A. Nigh, C. G. Kane, M. E. Sabatini, M. Kiebler, M. E. Greenberg, A brain-specific microRNA regulates dendritic spine development. *Nature* **439**, 283–289 (2006).

49. G. Polese, W. Winlow, A. Di Cosmo, Dose-dependent effects of the clinical anesthetic isoflurane on Octopus vulgaris: A contribution to cephalopod welfare. *J. Aquat. Anim. Health* **26**, 285–294 (2014).

50. A. Dobin, C. A. Davis, F. Schlesinger, J. Drenkow, C. Zaleski, S. Jha, P. Batut, M. Chaisson, T. R. Gingeras, STAR: Ultrafast universal RNA-seq aligner. *Bioinformatics* **29**, 15–21 (2013).

51. H. Li, Minimap2: Pairwise alignment for nucleotide sequences. *Bioinformatics* **34**, 3094–3100 (2018).

52. R. I. Kuo, Y. Cheng, R. Zhang, J. W. S. Brown, J. Smith, A. L. Archibald, D. W. Burt, Illuminating the dark side of the human transcriptome with long read transcript sequencing. *BMC Genomics* **21**, 751 (2020).

53. M. Tardaguila, L. de la Fuente, C. Marti, C. Pereira, F. J. Pardo-Palacios, H. Del Risco, M. Ferrell, M. Mellado, M. Macchietto, K. Verheggen, M. Edelmann, I. Ezkurdia, J. Vazquez, M. Tress, A. Mortazavi, L. Martens, S. Rodriguez-Navarro, V. Moreno-Manzano, A. Conesa, SQANTI: Extensive characterization of long-read transcript sequences for quality control in full-length transcriptome identification and quantification. *Genome Res.* **28**, 396–411 (2018).

54. X. Grau-Bové, I. Ruiz-Trillo, M. Irimia, Origin of exon skipping-rich transcriptomes in animals driven by evolution of gene architecture. *Genome Biol.* **19**, 135 (2018).

55. N. L. Barbosa-Morais, M. Irimia, Q. Pan, H. Y. Xiong, S. Gueroussov, L. J. Lee, V. Slobodeniuc, C. Kutter, S. Watt, R. Colak, T. Kim, C. M. Misquitta-Ali, M. D. Wilson, P. M. Kim, D. T. Odom, B. J. Frey, B. J. Blencowe, The evolutionary landscape of alternative splicing in vertebrate species. *Science* **338**, 1587–1593 (2012).

56. S. Memczak, M. Jens, A. Elefsinioti, F. Torti, J. Krueger, A. Rybak, L. Maier, S. D. Mackowiak, L. H. Gregersen, M. Munschauer, A. Loewer, U. Ziebold, M. Landthaler, C. Kocks, F. le Noble, N. Rajewsky, Circular RNAs are a large class of animal RNAs with regulatory potency. *Nature* **495**, 333–338 (2013).

57. H. Li, A statistical framework for SNP calling, mutation discovery, association mapping and population genetical parameter estimation from sequencing data. arXiv 1203.6372 [q-bio.GN] (28 March 2012).

58. I. Zarrella, K. Herten, G. E. Maes, S. Tai, M. Yang, E. Seuntjens, E. A. Ritschard, M. Zach, R. Styfhals, R. Sanges, O. Simakov, G. Ponte, G. Fiorito, The survey and reference assisted assembly of the *Octopus vulgaris* genome. *Sci. Data* **6**, 13 (2019).

59. S. H. Roth, E. Y. Levanon, E. Eisenberg, Genome-wide quantification of ADAR adenosine-to-inosine RNA editing activity. *Nat. Methods* **16**, 1131–1138 (2019).

60. W. Kang, Y. Eldfjell, B. Fromm, X. Estivill, I. Biryukova, M. R. Friedländer, miRTrace reveals the organismal origins of microRNA sequencing data. *Genome Biol.* **19**, 213 (2018).

61. B. M. Wheeler, A. M. Heimberg, V. N. Moy, E. A. Sperling, T. W. Holstein, S. Heber, K. J. Peterson, The deep evolution of metazoan microRNAs. *Evol. Dev.* **11**, 50–68 (2009).

62. Z. Gu, R. Eils, M. Schlesner, Complex heatmaps reveal patterns and correlations in multidimensional genomic data. *Bioinformatics* **32**, 2847–2849 (2016).

63. D. M. Emms, S. Kelly, OrthoFinder: Phylogenetic orthology inference for comparative genomics. *Genome Biol.* **20**, 238 (2019).

64. T. D. Wu, C. K. Watanabe, GMAP: A genomic mapping and alignment program for mRNA and EST sequences. *Bioinformatics* **21**, 1859–1875 (2005).

65. F. Sievers, A. Wilm, D. Dineen, T. J. Gibson, K. Karplus, W. Li, R. Lopez, H. McWilliam, M. Remmert, J. Söding, J. D. Thompson, D. G. Higgins, Fast, scalable generation of high-quality protein multiple sequence alignments using Clustal Omega. *Mol. Syst. Biol.* **7**, 539 (2011).

66. A. Deryckere, R. Styfhals, E. A. G. Vidal, E. Almansa, E. Seuntjens, A practical staging atlas to study embryonic development of *Octopus vulgaris* under controlled laboratory conditions. *BMC Dev. Biol.* **20**, 7 (2020).

67. P. Natsidis, P. H. Schiffer, I. Salvador-Martínez, M. J. Telford, Computational discovery of hidden breaks in 28S ribosomal RNAs across eukaryotes and consequences for RNA integrity numbers. *Sci. Rep.* **9**, 19477 (2019).

68. E. C. Winnebeck, C. D. Millar, G. R. Warman, Why does insect RNA look degraded? *J. Insect Sci.* **10**, 159 (2010).

69. L. Chen, S. J. Bush, J. M. Tovar-Corona, A. Castillo-Morales, A. O. Urrutia, Correcting for differential transcript coverage reveals a strong relationship between alternative splicing and organism complexity. *Mol. Biol. Evol.* **31**, 1402–1413 (2014).

70. T. W. Nilsen, B. R. Graveley, Expansion of the eukaryotic proteome by alternative splicing. *Nature* **463**, 457–463 (2010).

71. F. Li, L. Bian, J. Ge, F. Han, Z. Liu, X. Li, Y. Liu, Z. Lin, H. Shi, C. Liu, Q. Chang, B. Lu, S. Zhang, J. Hu, D. Xu, C. Shao, S. Chen, Chromosome-level genome assembly of the East Asian common octopus (*Octopus sinensis*) using PacBio sequencing and Hi-C technology. *Mol. Ecol. Resour.* **20**, 1572–1582 (2020).

72. M. D. Amor, S. R. Doyle, M. D. Norman, A. Roura, N. E. Hall, A. J. Robinson, T. S. Leite, J. M. Strugnell, Genome-wide sequencing uncovers cryptic diversity and mito-nuclear discordance in the *Octopus vulgaris* species complex. bioRxiv 573493 [**Preprint**]. 11 March 2019. https://doi.org/10.1101/573493.

73. M. D. Amor, M. D. Norman, A. Roura, T. S. Leite, I. G. Gleadall, A. Reid, C. Perales-Raya, C.-C. Lu, C. J. Silvey, E. A. G. Vidal, F. G. Hochberg, X. Zheng, J. M. Strugnell, Morphological assessment of the *Octopus vulgaris* species complex evaluated in light of molecular-based phylogenetic inferences. *Zool. Scr.* **46**, 275–288 (2017).

74. Y. Feng, C. L. Sansam, M. Singh, R. B. Emeson, Altered RNA editing in mice lacking ADAR2 autoregulation. *Mol. Cell. Biol.* **26**, 480–488 (2006).

75. O. Solomon, S. Oren, M. Safran, N. Deshet-Unger, P. Akiva, J. Jacob-Hirsch, K. Cesarkas, R. Kabesa, N. Amariglio, R. Unger, G. Rechavi, E. Eyal, Global regulation of alternative splicing by adenosine deaminase acting on RNA (ADAR). *RNA* **19**, 591–604 (2013).

76. P. Miura, S. Shenker, C. Andreu-Agullo, J. O. Westholm, E. C. Lai, Widespread and extensive lengthening of 3’ UTRs in the mammalian brain. *Genome Res.* **23**, 812–825 (2013).

77. V. Hilgers, M. W. Perry, D. Hendrix, A. Stark, M. Levine, B. Haley, Neural-specific elongation of 3’ UTRs during *Drosophila* development. *Proc. Natl. Acad. Sci. U.S.A.* **108**, 15864–15869 (2011).

78. R. Elkon, A. P. Ugalde, R. Agami, Alternative cleavage and polyadenylation: extent, regulation and function. *Nat. Rev. Genet.* **14**, 496–506 (2013).

79. S. Alon, E. Mor, F. Vigneault, G. M. Church, F. Locatelli, F. Galeano, A. Gallo, N. Shomron, E. Eisenberg, Systematic identification of edited microRNAs in the human brain. *Genome Res.* **22**, 1533–1540 (2012).

80. Y. Wan, K. Qu, Q. C. Zhang, R. A. Flynn, O. Manor, Z. Ouyang, J. Zhang, R. C. Spitale, M. P. Snyder, E. Segal, H. Y. Chang, Landscape and variation of RNA secondary structure across the human transcriptome. *Nature* **505**, 706–709 (2014).

81. N. Stoler, A. Nekrutenko, Sequencing error profiles of Illumina sequencing instruments. *NAR Genom. Bioinform.* **3**, lqab019 (2021).
